# Fecal filtrate transplantation as salvage therapy for fulminant *Clostridioides difficile* infection in adult hematological patients during chemotherapy-induced aplasia: a pilot experience

**DOI:** 10.1080/19490976.2026.2718620

**Published:** 2026-08-24

**Authors:** Dorina Korózs, Bálint Gergely Szabó, Gábor Apjok, Viktor Lakatos, Krisztina Jeszenszky, Katalin Kamotsay, Gabriella Hajbel-Vékony, Ákos Tóth, János Sinkó, Péter Reményi, Bálint Kintses

**Affiliations:** a Semmelweis University, Doctoral School of Clinical Medicine, Budapest, Hungary; b South Pest Central Hospital, National Institute of Hematology and Infectious Diseases, Budapest, Hungary; c Department of Haematology and Internal Medicine, Division of Infectology, Semmelweis University, Budapest, Hungary; d HUN-REN Biological Research Centre, National Laboratory of Biotechnology, Institute of Biochemistry, Synthetic and Systems Biology Unit, Szeged, Hungary; e National Center for Public Health and Pharmacy, Budapest, Hungary; f HUN-REN TKI, Budapest, Hungary

**Keywords:** Neutropenia, aplasia, phageome, virome, FMT, fecal microbiota transplantation, stool transplantation, stool supernatant, fecal filtrate, fecal filtrate transplantation

## Abstract

**Background:**

Fulminant *Clostridioides difficile* infection (CDI) in patients with hematologic malignancies during chemotherapy-induced aplasia carries high mortality and limited treatment options. Our objective was to evaluate fecal filtrate transplantation (FFT) as a salvage therapy for fulminant CDI during aplasia, and to explore whether donor-recipient phage dynamics may contribute to clinical response.

**Methods:**

We conducted a single-center, prospective, protocol-defined pilot case series study including consecutive adults with hematologic malignancies, chemotherapy-induced grade-4 aplasia and fulminant CDI, refractory to ≥5 d of high-dose oral vancomycin plus intravenous metronidazole and tigecycline. FFT was prepared from a single unrelated donor using sequential centrifugation and filtration, and administered via nasogastric tube in two doses. The primary outcome was sustained clinical cure, secondary outcomes included survival and adverse events, assessed at +14 and +30 d post-FFT. 16S rRNA gene sequencing was used to assess microbiome compositions, while viral metagenomics and *in vitro* propagation assays were employed to characterize donor-recipient phageome interactions.

**Results:**

Three patients with adverse-risk acute myeloid leukemia and fulminant CDI caused by genetically distinct *C. difficile* strains received FFT. All patients achieved clinical resolution by day +14, accompanied by improvements in abdominal distension and inflammatory markers. By day +30, one patient died from *Pseudomonas aeruginosa* septic shock, while two maintained remission with confirmatory follow-up. FFT was well tolerated, with no procedure-related immediate complications or FFT-attributable adverse events. Microbiome and phage profiling revealed heterogeneous responses, including shifts in bacterial community composition, with no clear evidence for a general role of donor-derived phages in CDI resolution. In contrast, we observed induction of prophages harbored by recipient-associated *Clostridium* species, which may have contributed to decolonization through stress-induced entry into the lytic cycle.

**Conclusions:**

FFT was feasible, with rapid sustained CDI resolution in hematologic patients with chemotherapy-induced aplasia.

**Trial registration:**

ClinicalTrials.gov identifier NCT07172191.

**Summary:**

Prospective single-center pilot study of fecal filtrate transplantation (FFT) for fulminant-refractory *C. difficile* infection in three aplastic adult hematologic patients. FFT was well tolerated, achieved rapid clinical cure, and showed heterogeneous microbiome and phageome modulation, potentially contributing to therapeutic effects.

## Graphical abstract

**Figure:**
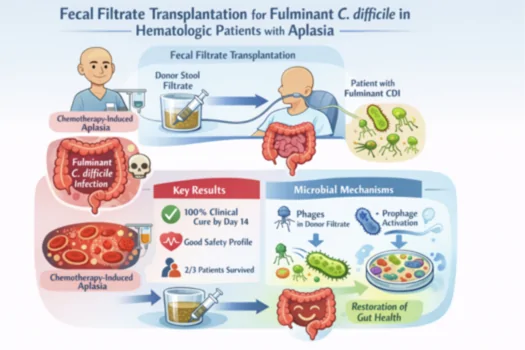

## Introduction

Over the past decade, *Clostridioides difficile* infection (CDI) has emerged as a significant nosocomial infection worldwide.^1-3^ CDI spans a broad clinical spectrum: approximately 2%–8% of cases progress to fulminant disease, which carries a high in-hospital mortality of 34%–57%.^4^ ^,^ ^5^ Patients receiving induction chemotherapy for acute leukemia or undergoing allogeneic hematopoietic stem-cell transplantation (HSCT) are at high-risk for infections due to chemotherapy-induced aplasia, which is frequently compounded by mucositis. In this setting, risk factors for CDI acquisition and severity, including prolonged hospitalization, broad-spectrum antibiotic exposure and neutropenia, are highly prevalent.^6^ Moreover, CDI is a leading cause of persistent diarrhea during treatment of hematologic malignancies, and in HSCT recipients, CDI has been linked to a risk for acute graft-versus-host disease with downstream immunosuppression and further infections.^7^

A summary of treatment options for fulminant CDI is provided in Supplementary Table 1. The only international guideline focused on hematologic patients notes limited options for severe-fulminant CDI, and calls for more data on FMT in neutropenia.^8^ Accordingly, FMT is not clearly positioned in this population, whereas in non-hematologic cohorts, it is considered as rescue therapy after standard treatment failure or when surgery is infeasible. Nonetheless, uptake in both settings is tempered by rare but serious complications, such as sepsis or aspiration pneumonia.^9^ The application of FMT in hematological patients with chemotherapy-induced aplasia may particularly raise specific concerns, as conventional FMT contains viable donor-derived microorganisms. In patients with profound neutropenia and mucosal barrier injury, this creates a theoretical risk of microbial translocation and invasive infection, even when rigorous donor screening is applied.

In our study, we describe the application of fecal filtrate transplantation (FFT) in the treatment of fulminant CDI among adult hematological patients during chemotherapy-induced aplasia for the first time. FFT was developed to mitigate the concern described above, by removing viable cells from the stool suspension through filtration. Consequently, the resulting fecal filtrate is devoid of bacterial, fungal, and host cells, while potentially retaining non-cellular biologically active constituents such as bacteriophages, metabolites, proteins, and other soluble molecules. Therefore, FFT may theoretically offer a lower risk of viable pathogen transfer than conventional FMT. We also aimed to assess whether phages originating from donor-derived stool filtrates or the recipient's microbiome could possibly contribute to clinical improvement post-transplantation.

## Methods

### Study design

We conducted a single-center, prospective, protocol-defined pilot case series study, enrolling consecutive adults with hematologic malignancies who developed chemotherapy-induced aplasia and fulminant CDI refractory to combination anti-CDI therapy. The study was performed at a national-referral center, in accordance with the Declaration of Helsinki and national ethics standards, with approval from the Scientific and Research Ethics Committee of the Hungarian National Medical Scientific Council (No.15754-3/2023/EÜIG). All participants provided written informed consent prior to inclusion.

### Patient identification and treatment

Chemotherapy-induced aplasia, febrile neutropenia, and mucositis were graded according to *European Society for Blood and Marrow Transplantation*, *European Society for Medical Oncology* and *National Comprehensive Cancer Network* guidance.^7^ ^,^ ^10^ CDI diagnosis, risk stratification and treatment followed *European Society of Clinical Microbiology and Infectious Diseases* (ESCMID) guidelines.^2^ ^,^ ^11^ Toxigenic *C. difficile* presence was detected from fresh-unformed stool by enzyme-immunoassay for glutamate dehydrogenase and toxins A/B (C. Diff Quik-Chek Complete, Techlab). Combination anti-CDI therapy consisted of oral vancomycin 500 mg QID, intravenous metronidazole 500 mg TID, and intravenous tigecycline 50 mg BID (after 100 mg loading-dose) for a minimum of 5 d for all patients, followed by FFT as the study intervention. Combination anti-CDI therapy was discontinued on the day of first FFT administration. Patients and care providers were informed about the intervention, but were blinded to filtrate preparation details.

### Fecal filtrate preparation

For all recipients, stool donations from a voluntary, single, unrelated, healthy 38-y-old Caucasian male donor, screened in accordance with international guidelines, including standardized medical history and laboratory/microbiological testing were used (Supplementary Files 1 and 2). Donor material was delivered to our institute within 30 min post-defecation. No financial compensation was provided.

The stool was stored in a sterile plastic container at +4 °C (+39.2 °F), and processed within 2 h post-defecation. The procedure was conducted in a biosafety cabinet (SafeFAST-Light, Faster). A weight-adjusted graft was prepared using 2 g stool/kg recipient body weight (120 g for Patients 1–2, 130 g for Patient 3). For every stool gram, 2 ml sterile 0.9% sodium chloride (NaCl) solution was added, and homogenized in a blender at maximum speed for 180 s (HR2052/00-450W, Philips). After coarse-sieving three times using a stainless-steel kitchen sieve, the mixture was centrifuged at 3455 × *g* at + 21 °C (+69.8°F) for three 30-min cycles (OHA-FC5718R Centrifuge, OHA-30314823 Swing-Out-Rotor, Ohaus). Between each cycle, ~95% of the supernatant was collected (ARK-MPIPE50, ARKA), and continued to the next cycle. The final supernatant was drawn into a 50 ml syringe (CH030502030PT, Chirana), and was passed through cellulose acetate membrane, polycarbonate housing syringe-filters of 0.45 µm and 0.22 µm (Filters [1470368, 1470367], Sterlitech). The final product was ultimately transferred into 50 ml syringes, and stored at −80 °C (−112 °F) until use after quality control (ULT-U250, NordicLab). The total administered FFT volume was 220 mL for Patients 1–2, and 240 mL for Patient-3.

### Quality control

Microbiological quality control of the final filtrate was performed using bacterial and fungal cultures. From this, a 0.5 McFarland inoculum was plated using a sterile inoculation loop (M4025-W, MTC-Bio) onto Columbia +5% sheep blood agar, Schäedler +5% sheep blood agar, Chocolate-PolyViteX chocolate agar, eosin-methylene blue agar, ChromID CPS-Elite agar, Sabouraud-dextrose agar (BioMérieux, France). Plates were incubated at +30° C (+86 °F) and +37 °C (+98.6 °F) under aerobic and anaerobic conditions for 48 h (184L-3541 Series-8000, Thermo-Scientific). As a positive control, unprocessed donor stool was plated, following identical procedures. Additionally, 10 ml of the final product was inoculated into an anaerobic blood culture bottle, and incubated aerobically at +37 °C (+98.6 °F) for 48 h in an automated incubator (BacT/ALERT-3D, bioMérieux). After 48 h, no colony growth was observed on any of the plates, whereas the positive control exhibited high colony-formation. Similarly, no signal was detected in the blood culture bottle after 48 h. For additional safety, fungal and anaerobic cultures were continued for 7 d, without any detectable growth.

### Method of administration

FFT was administered in two doses ~5 d apart via a single-lumen, 14-Fr/120-cm, Levin-type nasogastric tube (Changzhou Weite Medical Equipment Co.), after metoclopramide premedication, followed by sterile saline flushing. For thawing, a blood warmer was used with 1-h heating-cycle (SAHARA-III/230V, SARSTEDT). Pre-FFT stool samples were collected from all patients.

### Patient follow-up and outcomes

Patients were followed daily *per protocol* for +30 d post-FFT. Clinical assessments included abdominal circumference, stool frequency and consistency, documentation of adverse events graded by *Common Terminology Criteria for Adverse Events (CTCAE) v6.0* (https://dctd.cancer.gov/research/ctep-trials/for-sites/adverse-events). Laboratory parameters included complete blood count, serum C-reactive protein (CRP), procalcitonin (PCT), albumin, creatinine. Contrast-enhanced abdominal computed tomography (CT) was performed on FFT day, and day + 10 ± 2. The primary outcome was sustained CDI cure, per ESCMID definitions.^11^ Secondary outcomes were survival and adverse events. Outcomes were assessed at days +14 and +30, after the first FFT dose. For reporting, we adhered to the TIDieR Checklist and CONSORT Statement (Supplementary Files 3 and 4).

### Characterization of *C. difficile* strains

*C. difficile* isolates were characterized by multiplex-PCR targeting toxin A (*tcdA*), toxin B (*tcdB*), and binary toxin (*cdtA/cdtB*) genes. PCR ribotyping was performed by capillary-gel electrophoresis using the primers described by Bidet et al., with ribotype assignment via WEBRIBO database (https://webribo.ages.at/).^12^ ^,^ ^13^ For whole-genome sequencing, libraries were prepared with the Illumina DNA-Prep library preparation kit, and sequenced with Illumina MiSeq 2 × 150 bp paired-end kit (Illumina, San Diego, US). Using SeqSphere +10.0.5 software (Ridom, Münster, Germany), we conducted genomic data analysis and *de novo* assembly by SKESA. Clonal relationships were investigated by multilocus sequence typing (MLST), and core genome MLST (cgMLST) stable scheme of SeqSphere+.

### 16S rRNA sequencing and bioinformatics of microbiome analysis

16s rRNA sequencing was performed as described earlier by our group.^14^ The following five unmanipulated stool samples were prepared for sequencing: (i) donor fecal sample [donor], plus each patient's (ii) pre-FFT fecal samples [Patient-1_pre_FFT, Patient-2_pre_FFT, Patient-3_pre_FFT], (iii) post-FFT fecal samples at day +14 [Patient-1_post_FFT_d14, Patient-2_post_FFT_d14, Patient-3_post_FFT_d14], and (iii) post-FFT fecal samples at day +30 [Patient-1_post_FFT_d30, Patient-3_post_FFT_d30].

Raw paired-end sequencing data (Illumina MiSeq, 2 × 300 bp) targeting the V3-V4 hypervariable region of the 16S rRNA gene were processed and analyzed using the QIIME 2 software package (version 2024.10).^15^ Quality control, denoising, and chimera filtering were performed using the DADA2 plugin. Alpha rarefaction curves for the 16S rRNA gene amplicon dataset were generated in QIIME2 from the unrarefied ASV feature table (Supplementary Figure 1). Based on the visual inspection of the Phred quality score profiles, forward and reverse reads were truncated at 270 and 220 bases, respectively. This truncation strategy successfully removed low-quality base calls at the 3′ ends while maintaining a sufficient overlap for paired-end read merging. The resulting Amplicon Sequence Variants (ASVs) were used for all downstream analyses. For phylogenetic diversity analyses, a rooted phylogenetic tree was constructed from the representative ASV sequences. To account for uneven sequencing depths across samples, the ASV feature table was rarefied to an even sampling depth of 4290 reads per sample. This threshold was determined based on the sample with the lowest read count, ensuring the retention of all samples in the final analysis. Alpha diversity was evaluated using Shannon entropy, Faith's Phylogenetic Diversity, Pielou's Evenness, and the number of observed features by ASV richness (Supplementary Table 2). Beta-diversity analyses were performed in QIIME2 at the ASV level using the uncollapsed feature table rarefied to 4290 reads per sample (Supplementary Table 3). Bray–Curtis, Jaccard, weighted UniFrac, and unweighted UniFrac distance matrices were calculated; UniFrac analyses additionally used the rooted ASV phylogeny (Supplementary Figures 2 and 3, Supplementary Tables 4 and 5, Supplementary File 5).

**Figure 1.**
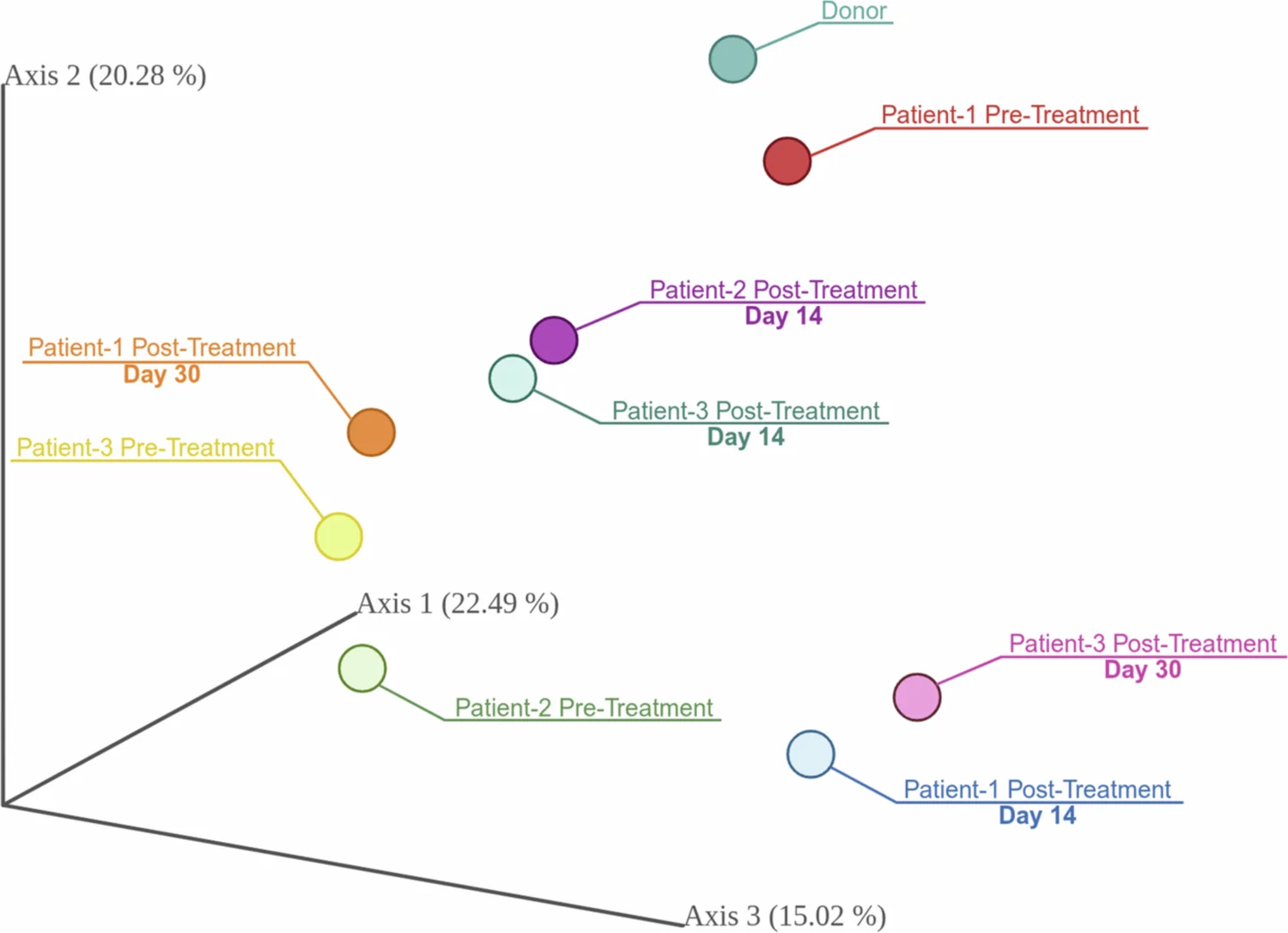
Principal coordinates analysis of gut microbiota compositional dynamics post-transplantation. The plot represents beta diversity based on the Bray–Curtis dissimilarity matrix, evaluating the structural differences and relative abundances of the identified ASVs across the microbial communities. Individual data points represent fecal samples; the percentage of variance explained by each principal coordinate is indicated on the respective axes (PC1: 22.49%, PC2: 20.28%, and PC3: 15.02%).

**Figure 2.**
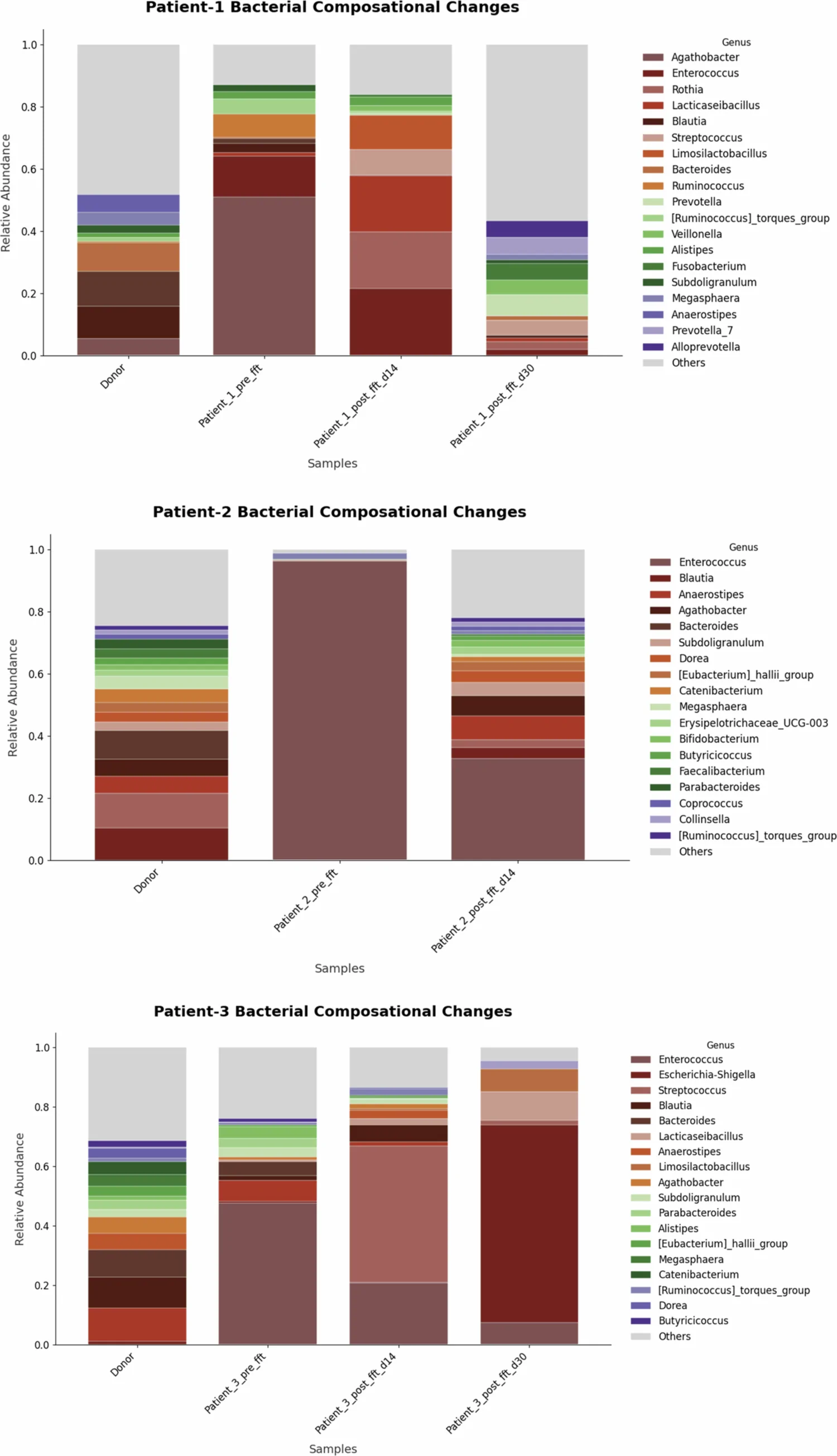
Taxonomic composition of the microbial communities across donor and patient samples. Stacked bar plots illustrate the relative abundance of the top 20 most highly abundant bacterial taxa, resolved to the genus level (QIIME2 Level-6), for each sample. All remaining low-abundance taxa were aggregated into the “Others” category (light gray). Donor samples used for 16S rRNA gene sequencing represented distinct donor stool/source samples, rather than aliquots of a single identical donation.

The resulting beta-distances were visualized using Principal Coordinates Analysis (PCoA) to assess the compositional shifts (Figure 1). Taxonomic classification of the representative sequences was performed using the q2-feature-classifier plugin. The classifier was trained on the SILVA reference database (release 138), specifically optimized for the 16S rRNA V3-V4 region using the scikit-learn (version 1.4.2) framework. Relative abundance of bacterial taxa (Supplementary Table 6) was visualized at various taxonomic levels (Figure 2).

### *In vitro* modeling of donor-derived phage dynamics

**Figure 3.**
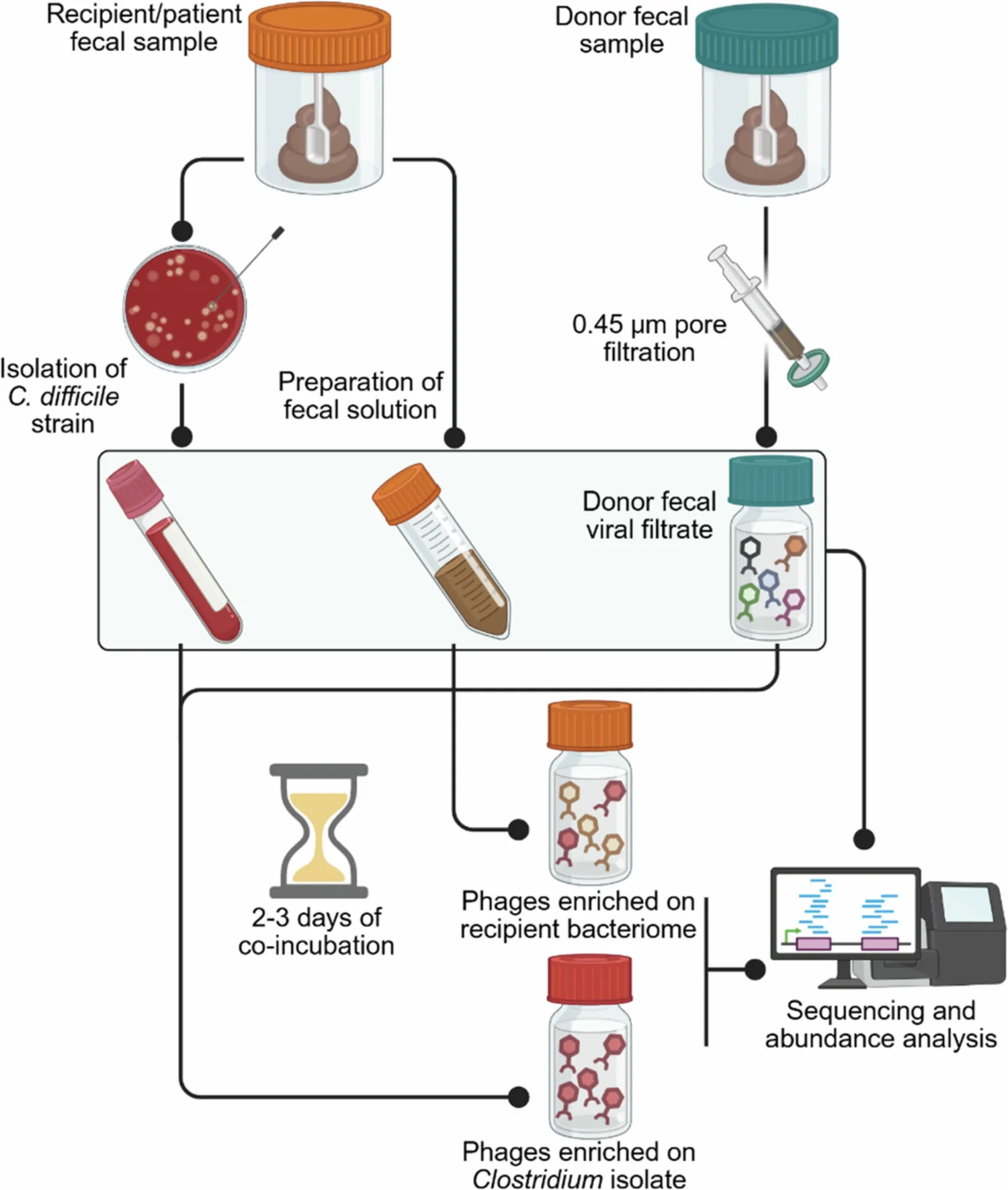
Concept figure of the applied experimental pipeline to assess changes in phageome due to application of donor stool supernatant to *C. difficile* isolates and recipient fecal solutions.

To explore whether donor-derived phages or other filtrate components contribute to clinical improvement after FFT, we adapted the protocol of Fitzgerald et al.^16^ Briefly, we cultivated each patient's pre-FFT fecal material and *C. difficile* isolate with and without donor stool filtrate, under anaerobic conditions (Figure 3). Donor Filtrates, serving as baseline inputs for subsequent propagation experiments, were prepared as described earlier. To assess phage propagation targeting *C. difficile* and/or other commensals/pathogens within the host, we performed two parallel assays with negative controls: (i) isolate-based propagation assay, where experimental samples of Donor Filtrate (500 µL) were mixed with one of three *C. difficile* isolates (500 µL) +1 mL medium, and control samples, with each *C. difficile* isolate mixed with an equal volume of medium, and (ii) whole-stool propagation assay, where experimental samples were composed of Donor Filtrates mixed with dissolved pre-FFT fecal material, while control samples contained patient stool + medium. Cultures were incubated for 48 h under *C. difficile*-specific anaerobic conditions, according to the in-house protocol of the Hungarian National Center for Public Health and Pharmacy.

**Figure 4.**
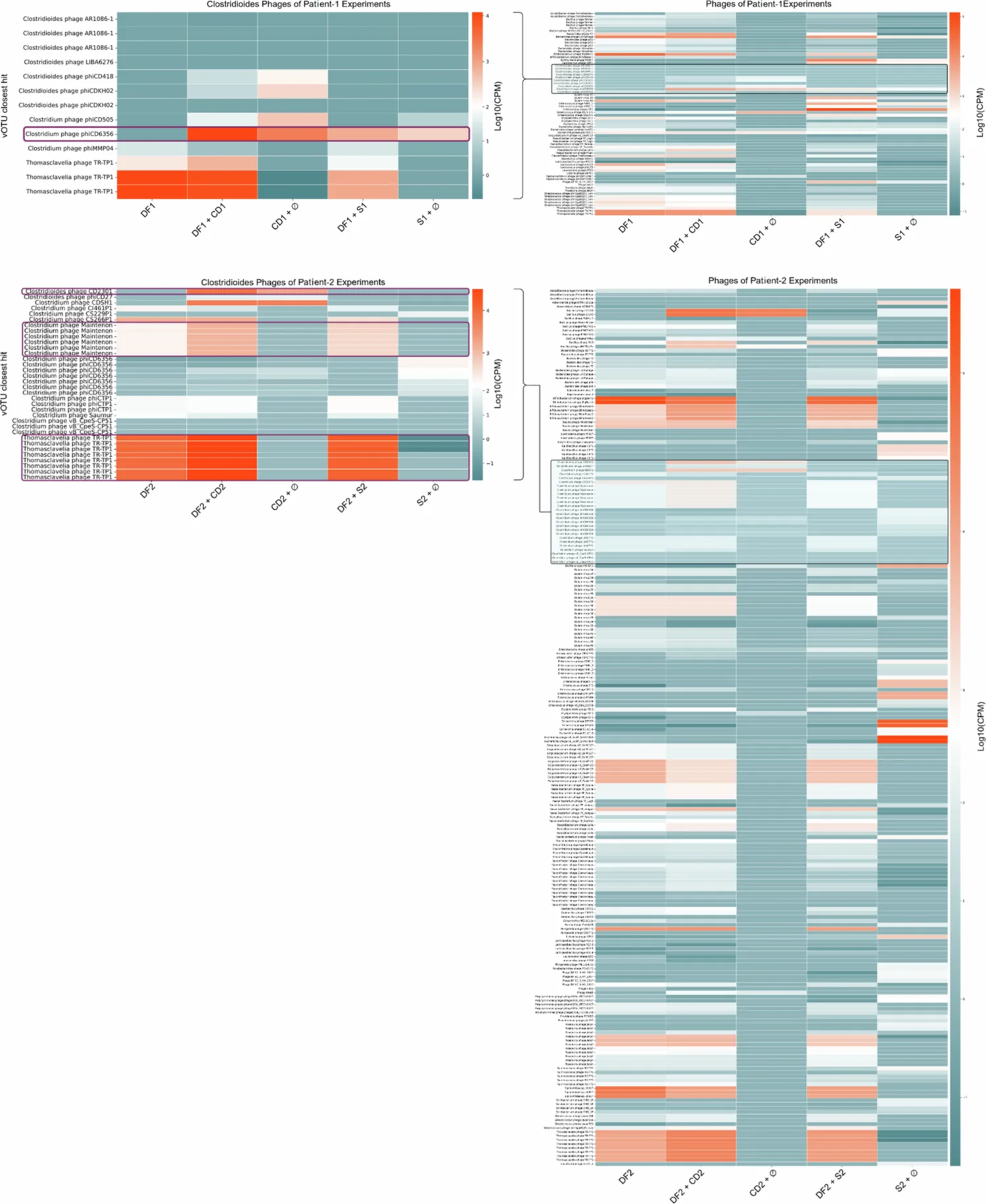
CPM based abundance values of predicted contigs visualized on heatmaps of Patient-1, Patient-2, and Patient-3, respectively. *Clostridioides* sections are enlarged for better visibility. Enriched, donor derived *Clostridioides* phages are highlighted with purple brackets.

After incubation, viral filtrates were generated from each culture, and next-generation sequencing was applied to identify viral operational taxonomic units (vOTUs) of phage origins, selectively amplified in donor material presence. The following five viral filtrates were prepared for sequencing from each sample, using the protocol described above: (i) Donor Filtrates alone (baseline phage abundance levels; DF1/2/3 + Ø), (ii) Donor Filtrates + Patient-1/2/3 *C. difficile* isolates (*C. difficile*-specific phage propagation; DF1/2/3 + CD1/2/3), (iii) Patient-1/2/3 *C. difficile* isolates + medium (control of contamination and baseline prophage production, CD1/2/3 + Ø), (iv) Donor Filtrates + Patient-1/2/3 pre-FFT stool (donor phageome dynamics against recipient bacteria; DF1/2/3 + S1/2/3), (v) Patient-1/2/3 pre-FFT stool + medium (baseline recipient growth dynamics, S1/2/3 + Ø). By sequencing *C. difficile* isolate genomes along with viral and bacterial fractions of donor stool, we determined whether amplified phages were donor- or recipient-derived, and identified their bacterial origins (Figure 4). Samples were sequenced using Illumina Nextera XT (paired-end, 2 × 150 bp; 10 M reads/sample), a detailed description is provided in the Supplementary File 6. Where relevant, phage lifestyle was predicted using BACPHLIP and completeness was calculated via checkV.^17^ ^,^ ^18^

## Results

**Figure 5.**
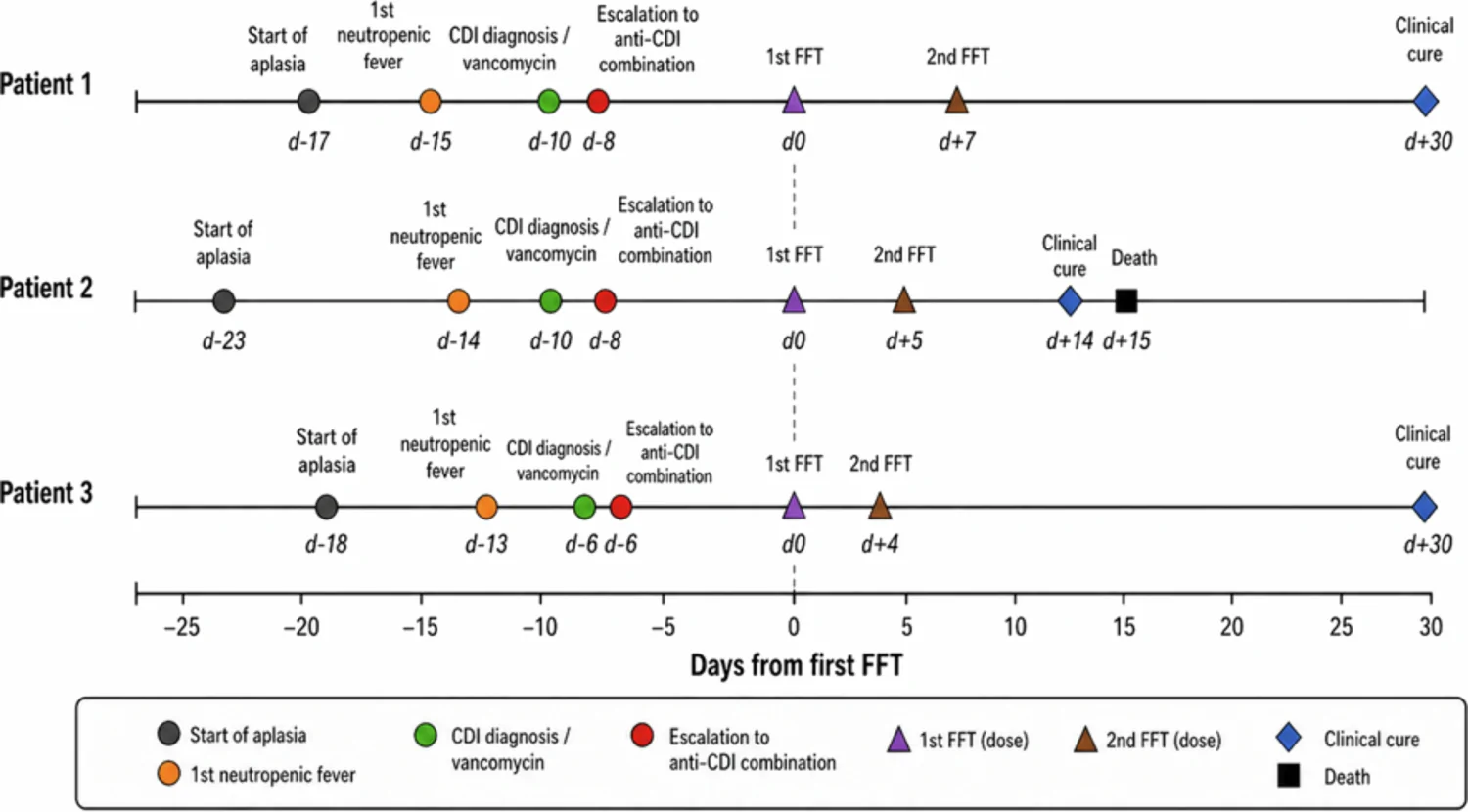
Individual patient timelines, referenced to first FFT as Day 0.

**Figure 6.**
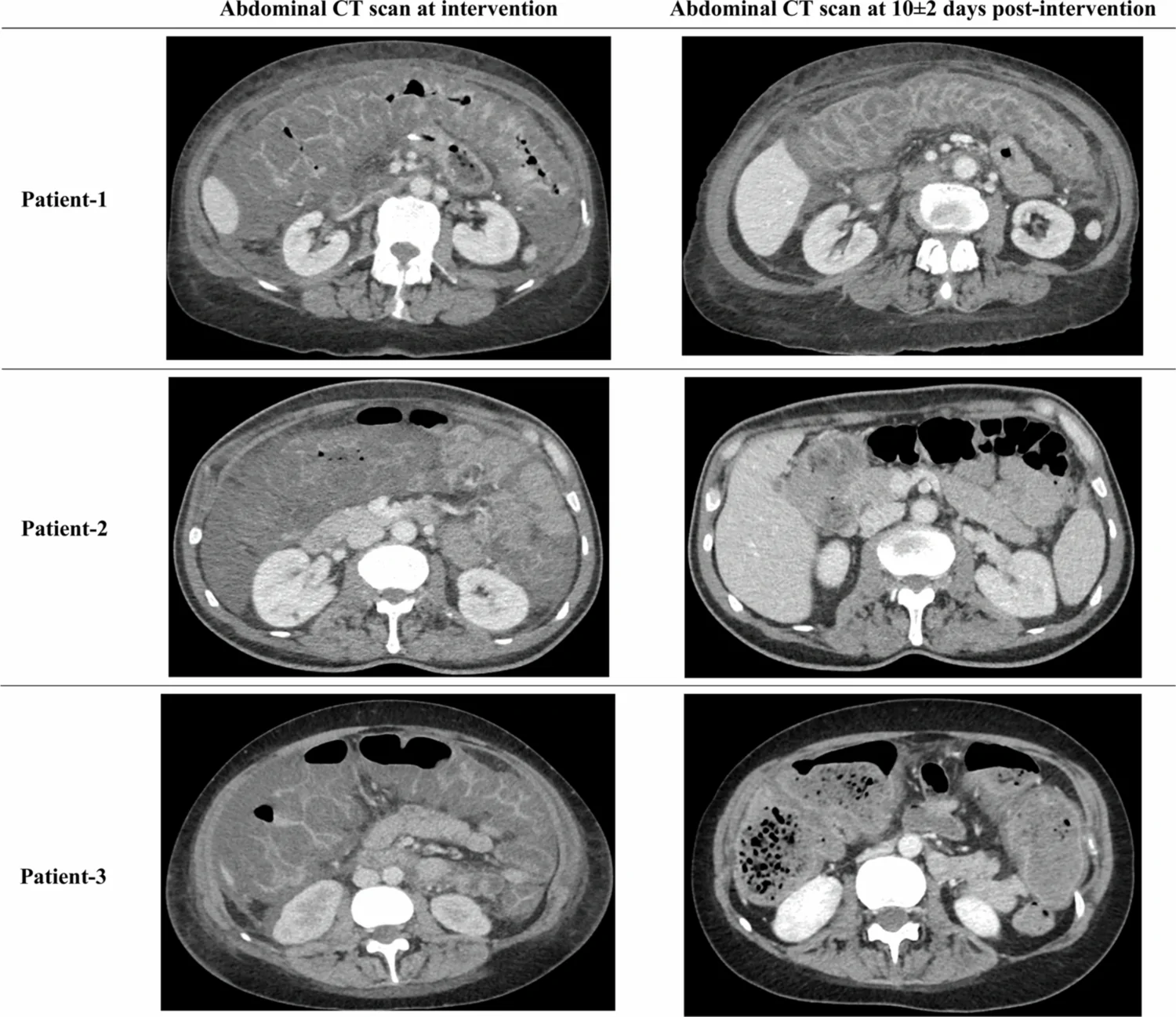
Representative contrast-enhanced abdominal CT images acquired in the horizontal plane at the level of the transverse colon in study participants.

**Figure 7.**
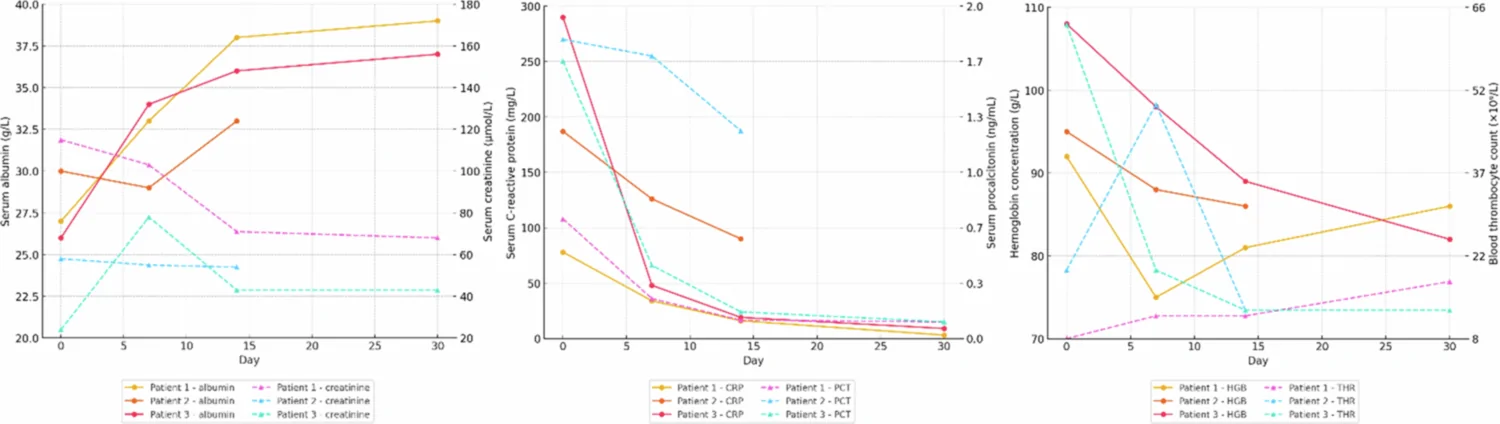
Selected laboratory parameters of study patients. Day 0 marks the day of intervention in each case. CRP: C-reactive protein, HGB: hemoglobin, PCT: procalcitonin, THR: thrombocyte.

**Figure 8.**
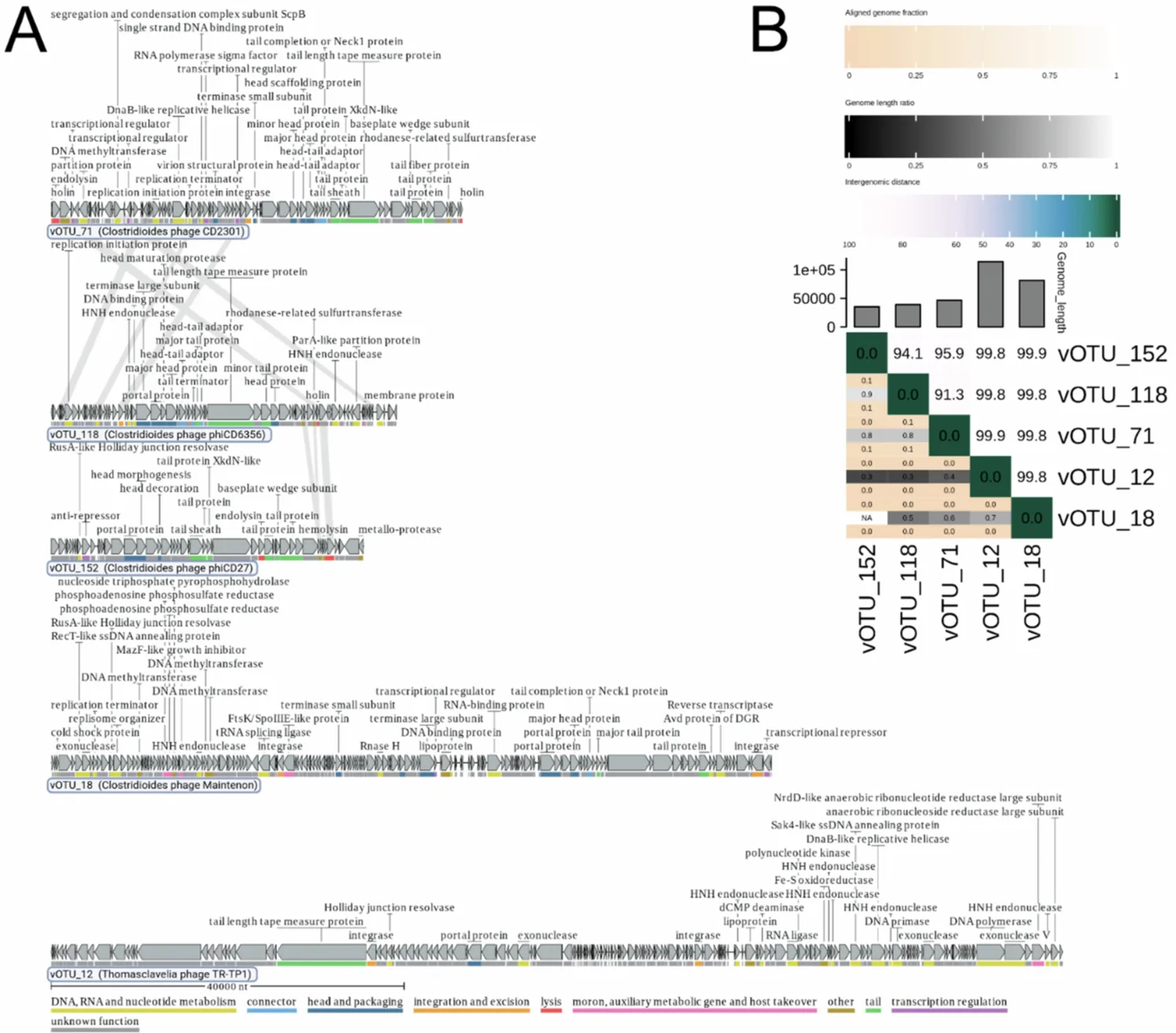
Comparative analysis of representative viral operational taxonomic units (vOTUs) using LoVIS. (A) Annotated genome maps of six Clostridioides-targeting vOTUs were aligned with LoVIS4U to identify shared gene synteny and functional modules (as described by Egorov et al., NAR Genom Bioinform, 2025). Genes are color-coded by functional category, with core structural and replication-associated proteins conserved across most genomes. (B) Pairwise comparisons of vOTUs are shown via a heatmap summarizing intergenomic distances, alignment fractions, genome length ratios, and total alignment lengths with VIRIDIC (as described by Moraru et al., Viruses, 2020).

Three patients were enrolled in the study. Baseline characteristics are summarized in Table 1. Individual patient timelines are presented in Figure 5, CT imaging findings in Figure 6, laboratory trends in Figure 7, 16S rRNA gene sequencing-based analysis of the bacterial microbiome in Figure 2, and metagenomic profiling of the viral fraction in Figures 4 and 8.

**Table 1.** Clinical characteristics and outcomes of patients included in the study.

Characteristics	Patient-1	Patient-2	Patient-3
Age (y), sex, race	60, female, Caucasian	64, male, Caucasian	19, female, Caucasian
Underlying non-hematological disease	Vater papilla adenocarcinoma (in complete remission for ≥5 y)	None	None
Hematologic characteristics:			
Underlying hematological malignancy	CMMoL/AML	De novo AML	MDS/AML
Risk stratification of hematological malignancy	Adverse	Adverse	Adverse
Disease status before current hematological therapy	First relapse	Newly diagnosed	Complete remission
Current hematological therapy	FLAG-M	CLA-Ven	Thio-Bu-Flu
Current treatment intention	Salvage	Remission induction	Myeloablative conditioning
Neutropenia duration, before diagnosis of CDI (d)	7	13	12
Mucositis after current treatment, before diagnosis of CDI	No	No	Yes (Grade 4)
ECOG performance status at diagnosis of CDI	4	3	4
Characteristics of the causative *C. difficile* strain:			
tcdA, tcdB, cdtA/cdtB toxin PCR status	+, +, −/−	+, +, +/+	+, +, −/−
Sequence type	ST17	ST1	ST3
Complex type	CT7513	CT11377	CT10432
Ribotype	RT638	RT181	RT451
Combination anti-CDI treatment	Oral vancomycin 500 mg QID + intravenous metronidazole 500 mg TID + intravenous tigecycline 50 mg BID, after 100 mg loading dose		
Immunosuppressive medications on the day of first FFT dose	None	None	Methylprednisolone, ruxolitinib, tacrolimus
Parenteral fluid replacement therapy on the day of first FFT dose	Yes	Yes	Yes
Parenteral nutrition administration on the day of first FFT dose	Yes	Yes	Yes
Intravenous albumin administration on the day of first FFT dose	Yes	Yes	Yes
Timing of first FFT dose after diagnosis of CDI	On day 8	On day 8	On day 6
Total body weight on the day of first FFT dose (kg)	60	61	65
Total volume of administered FFT doses (ml)	2 × 110	2 × 110	2 × 120
ECOG performance status during follow-up:			
Day of first FFT dose	4	4	4
FFT + 7 d	4	4	4
FFT + 14 d	3	4	3
FFT + 30 d	2	n.a.	2
Abdominal pain on the visual analog scale during follow-up:			
Day of first FFT dose	10	9	10
FFT + 7 d	8	6	7
FFT + 14 d	5	5	4
FFT + 30 d	0	n.a.	1
Bowel movements per day:			
Day of first FFT dose	10	13	17
FFT + 7 d	5	9	7
FFT + 14 d	3	5	4
FFT + 30 d	2	n.a.	2
Median Bristol stool score:			
Day of first FFT dose	7	7	7
FFT + 7 d	5	5	6
FFT + 14 d	4	4	4
FFT + 30 d	4	n.a.	4
Abdominal circumference (centimeter):			
Day of first FFT dose	108	89	97
FFT + 7 d	100	87	92
FFT + 14 d	96	83	89
FFT + 30 d	84	n.a.	85
Outcomes at FFT + 14 d:			
Sustained cure of CDI	Yes	Yes	Yes
Patient survival	Yes	Yes	Yes
Adverse events	No	No	No
Outcomes at FFT + 30 d:			
Sustained cure of CDI	Yes	n.a.	Yes
Patient survival	Yes	No^a^	Yes
Adverse events	No	n.a.	No

AML: acute myeloid leukemia, BID: *bis in die* (twice daily), CDI: *Clostridioides difficile* infection, CLA-Ven: cladribine + cytosine-arabinoside + venetoclax, CMMoL: chronic myelomonocytic leukemia, CT: complex type, ECOG: Eastern Cooperative Oncology Group, FFT: fecal filtrate transplantation, FLAG-M: fludarabine + cytosine arabinoside + filgrastim + mitoxantrone, HSCT: hematopoietic stem cell transplantation, MDS: myelodysplastic syndrome, n.a.: not applicable, QID: *quater in die* (four times daily), RT: ribotype, ST: sequence type, Thio-Bu-Flu: Thiotepa + busulphan + fludarabine, TID: *ter in die* (three times daily), WGS: whole genome sequencing.

^a^ Due to septic shock caused by *Pseudomonas aeruginosa*, unrelated to the intervention.

16S rRNA gene sequencing of the donor fecal sample [donor] identified 110 ASVs, revealing a balanced microbiome dominated by obligate anaerobic *Firmicutes*, particularly members of the *Clostridia* class and *Lachnospiraceae* family, with adequate representation of *Bacteroidales* and very low relative abundance of *Enterococcus* and *Enterobacteriaceae* (Figure 2, Supplementary Tables 2–6). In parallel, virome analysis [DF1/2/3 + Ø] identified a diverse donor viral community (Supplementary Figures 1–7). Overall, this composition is mostly consistent with a non-dysbiotic gut microbiome profile with high anaerobic diversity.

### Patient-1

A 60-y-old Caucasian woman with relapsed acute myeloid leukemia (AML) transformed from chronic myelomonocytic leukemia developed grade-4 aplasia after FLAG-M reinduction (fludarabine, cytarabine, mitoxantrone). On the second day of aplasia, neutropenic fever developed, requiring empirical cefepime. Subsequently, *Enterococcus gallinarum* bloodstream-infection was confirmed, leading to linezolid escalation. On the seventh day of aplasia, diarrhea developed and CDI was confirmed. Standard-dose oral vancomycin monotherapy was initiated, but progressive abdominal distension and pain developed, fulfilling criteria for fulminant CDI with toxic megacolon on CT. Despite the initiation of combination anti-CDI treatment two days later, clinical deterioration continued with worsening renal dysfunction and abdominal distension. FFT was administered on after eight days of combined anti-CDI therapy and was followed by rapid clinical improvement, including decreasing abdominal distension, reduced stool frequency, declining serum CRP and renal function recovery. A second FFT dose was administered seven days after the first dose (day FFT + 7). Sustained CDI cure was achieved by day FFT + 14 and maintained through day FFT + 30, with no documented adverse events.

Patient-1 exhibited a dysbiotic microbiome prior to FFT, characterized by low species diversity (44 ASV) and a disproportionately high abundance of a single anaerobic taxon, *Agathobacter*, alongside co-dominance of *Enterococcus* (Figure 2, Supplementary Table 2). Following FFT, the microbiome by day FFT + 14 showed a transient expansion of *Lactobacillales/Enterococcus,* and a reduction in obligate anaerobes, reflecting a shift away from a donor-like community structure (Figure 1, Supplementary Tables 3–6). By day FFT + 30, microbial diversity markedly increased, with the number of ASVs rising to 194, indicating an ecological expansion likely driven by previously low-abundance, endogenous taxa. *In vitro* analyses of [DF1 + CD1] and [DF1 + S1] samples showed no enrichment of donor-derived *C. difficile*-specific phages in Patient-1. Instead, donor supernatant exposure was associated with a marked increase in the abundance of a prophage encoded within the *C. difficile* genome (vOTU_118; Figure 4, Supplementary Figure 4), consistent with prophage induction. vOTU_118, classified as a temperate phage (please refer to the Methods), showed the highest similarity to *Clostridium* phage phiCD6356 (~91% similarity, with 100% genome completeness predicted by CheckV; Supplementary Tables 7 and 8), a temperate phage first isolated after mitomycin-C induction of a lysogenic *C. difficile* strain, and capable of productive replication associated with host-cell lysis.^19^ As prophage induction in *C. difficile* can be triggered by physiological stressors, including secondary bile acids and antibiotic exposure, components of the donor-derived fecal filtrate may have contributed to the observed increase in vOTU_118 abundance.^20^ ^,^ ^21^ However, our data do not allow us to determine whether prophage induction contributed to the clinical response beyond the established effects of secondary bile acids on *C. difficile* growth and survival. Donor supernatant treatment also affected recipient-derived prophages in several non-*Clostridium* taxa, with Enterococcus-associated prophages vOTU_3 (*Enterococcus* phage EF1) and vOTU_352 (*Enterococcus* phage 4995_1) being particularly abundant in [DF1 + S1].

### Patient-2

A 64-y-old Caucasian man with newly diagnosed, adverse-risk AML developed grade-4 aplasia during CLA-Ven induction (cladribine, low-dose cytarabine, venetoclax). On the ninth day of aplasia, neutropenic fever developed, requiring administration of empirical cefepime. On the 13th day of aplasia, CDI was confirmed after onset of abdominal pain and diarrhea. Despite standard-dose vancomycin, the patient rapidly progressed to fulminant disease with pancolitis and ascites. Combination anti-CDI therapy was escalated two days later, but symptoms and inflammatory markers continued to worsen. FFT was administered after eight days of combined anti-CDI therapy, after which abdominal distension stabilized and serum CRP began to decline. A second FFT was given five days after the first dose (day FFT + 5), and CDI cure was achieved by day FFT + 14. The patient subsequently developed Pseudomonas aeruginosa septic shock on the next day, and died despite appropriate treatment. The blood culture isolate was identical to the one previously identified from the patient's surveillance cultures, and was not detected by culturing methods in donor samples.

Patient-2 exhibited a highly dysbiotic microbiome prior to FFT, characterized by very low diversity, with 22 ASVs and near-complete dominance of *Enterococcus*, and minimal representation of obligate anaerobic taxa (Figure 2, Supplementary Table 2). Following FFT, the microbiome at day 14 showed a marked restructuring toward an anaerobe-rich community, with substantial expansion of several *Clostridia* and *Lachnospiraceae* genera. This was accompanied by a pronounced increase in species richness, rising to 87 ASVs, indicating substantial ecological recovery. Beta-diversity analysis further demonstrated a clear shift toward the donor microbiome profile (Figure 1, Supplementary Tables 3–6). The increased abundance of these obligate anaerobes is consistent with the restoration of a microbial community capable of enhanced colonization resistance against *C. difficile.*

In contrast to the observation with Patient-1's phageome profiles, samples from Patient-2 exhibited broad suppression of multiple recipient-derived prophage vOTUs compared to controls (Figure 4, Supplementary Figure 4). Prominently, vOTU_120 (*Escherichia* phage vB_EcoM_SHAK7854) declined from the highest baseline abundance in [S2 + Ø], to minimal/zero levels in post-treatment [DF2 + S2]. Concurrently, enrichment of two donor-derived *Clostridium*-related phages was observed: vOTU_18 (*Clostridium* phage Maintenon) and vOTU_12 (*Thomasclavelia* phage TR-TP1). Interestingly, vOTU_18 was traced to a donor bacterium identified as *Agathobacter rectalis*, representing an instance of a prophage potentially targeting a cross-genus taxon.

### Patient-3

A 19-y-old Caucasian woman in complete remission from adverse-risk AML secondary to myelodysplastic syndrome underwent haploidentical allo-HSCT, following myeloablative conditioning. On the fifth day of aplasia, she developed grade-4 mucositis and neutropenic fever, requiring empirical cefepime. Subsequent blood cultures yielded *Granulicatella adiacens*. On the 11th day of aplasia, vancomycin-resistant *Enterococcus faecium* bloodstream-infection was documented behind a recurring fever, leading to escalation with linezolid. One day later, the patient developed hemodynamic instability and near-complete bowel obstruction. Abdominal CT revealed pancolitis with features consistent with pseudomembranous colitis. Despite the immediate initiation of anti-CDI combination therapy and its continuation for 6 d, painful abdominal distension and renal dysfunction progressed. After FFT, the patient improved clinically with decreasing abdominal distension and normalization of stool frequency, and follow-up CT showed marked colitis regression. A second dose was administered four days after the first FFT (day FFT + 4). Sustained CDI cure was achieved by day FFT + 14, and maintained through day FFT + 30 without adverse events.

Patient-3 exhibited a diverse pre-treatment microbiome (129 ASVs) with a composition resembling the donor profile, despite a high abundance of Enterococcus (~47.7%) (Figure 2, Supplementary Table 2). Following FFT, the microbiome at day 14 displayed a mixed and unstable profile, characterized by dominance of *Streptococcus* (~45.9%) and *Enterococcus* (~20.8%), alongside partial recovery of anaerobic taxa such as *Blautia* and *Anaerostipes*, while *C. difficile* remained detectable at low abundance. By day 30, *C. difficile* ASV disappeared, while the microbiome underwent a marked collapse in diversity, decreasing to 44 ASVs, and diverged substantially from the donor (Figure 1, Supplementary Tables 3–6). This was characterized by an expansion of *Escherichia/Shigella* (~66.6%). Overall, findings indicate a transient and incomplete microbiome restructuring following FFT, without stable establishment of anaerobic taxa associated with secondary bile acid metabolism and colonization resistance against *C. difficile*.

In Patient-3, *in vitro* donor filtrate exposure produced minimal overall changes in the phageome profiles (Figure 4, Supplementary Figure 4). Nonetheless, a modest induction of a *Clostridioides* prophage (vOTU_152, *Clostridium* phage phiCD27) present in the Patient-3's *C. difficile* genome was observed. Notably, *C. difficile* was detectable in the patient microbiome even at 14 d post-FFT, which resolved by day 30. Donor filtrate exposure in the [DF3 + CD3], but not [DF3 + S3] also led to selective repression of several *Lacticaseibacillus* prophages (vOTU_349, vOTU_103, vOTU_230).

## Discussion

### Clinical findings of our study

To our knowledge, this is the first report of fecal filtrate transplantation as a therapeutic approach for fulminant *C. difficile* infection in adults with hematologic malignancies during chemotherapy-induced aplasia. Three patients with acute leukemia or allo- HSCT developed fulminant CDI during grade-4 aplasia, caused by genetically distinct *C. difficile* strains, and deteriorated despite a guideline-based combination therapy of high-dose oral vancomycin, intravenous metronidazole and tigecycline. Following FFT, all patients exhibited a rapid and sustained clinical improvement including reduced abdominal distension and stool frequency. Additionally, serum C-reactive protein, creatinine and albumin levels showed a trend toward normalization. Despite distinct *C. difficile* strain lineages, including variations in toxin content, sequence types, ribotypes and complex classification, all patients demonstrated clinical response after FFT, supporting a potentially strain-agnostic effect.

Our 16S rRNA gene sequencing analyses revealed heterogeneous microbiome responses to FFT, despite consistent clinical resolution of CDI. All patients exhibited high baseline abundance of *Enterococcus* prior to FFT, a genus that enhances the fitness and pathogenesis of *C. difficile.* ^22^ Following FFT, we observed variable microbiome restructuring, including expansion of obligate anaerobic *Firmicutes* such as *Clostridia* and *Lachnospiraceae* in some cases, consistent with a shift toward a community with increased potential for secondary bile acid metabolism.^23^ In other cases, restructuring was partial or unstable, including increased diversity without clear convergence toward the donor profile or only transient recovery of anaerobic taxa. Collectively, these findings suggest that while re-establishment of anaerobic, bile acid–transforming microbiota may contribute to colonization resistance against *C. difficile*, which is consistent with previous findings.

### The phageome: a key to clinical cure?

In Patient-1, the absence of donor-derived *Clostridioides* phage enrichment was accompanied by induction of the prophage vOTU_118 (*Clostridium* phage phiCD6356) encoded in the *C. difficile* genome. This finding suggests that donor filtrate exposure, possibly through possibly through metabolite-mediated bacterial stress and microbiome restructuring, may have promoted prophage activation. Although this could theoretically contribute to *C. difficile* suppression through bacterial host-cell lysis, our data do not allow us to determine its direct contribution to clinical resolution.^21^ Widespread prophage induction was also observed in non-*Clostridioides* taxa, particularly *Enterococcus*-associated prophages vOTU_3 (*Enterococcus* phage EF1) and vOTU_352 (*Enterococcus* phage 4995_1). Given the synergy between *Enterococcus* and *Clostridioides* pathogenesis, prophage-driven destabilization of *Enterococcus* populations may have also supported clinical improvement.^22^ In Patient-2, donor filtrate exposure led to suppression of prophage production across several taxa in specific vOTUs, including *Escherichia, Klebsiella, Enterococcus,* and *Akkermansia*. This suppression may reflect direct phage-phage competition, quorum-sensing interference, or repression via unknown donor-derived components.^24-26^ Patient-2 also exhibited enrichment of two donor-derived *Clostridium* phages, vOTU_18 (*Clostridium* phage Maintenon) and vOTU_12 (*Thomasclavelia* phage TR-TP1). While vOTU_12 was predicted to infect *Thomasclavelia*, a genus previously classified as *Clostridium ramosum*, its proteomic profile aligns with *Clostridium* phages, supporting its inclusion within this functional group. Even more strikingly, vOTU_18 appears to be carried by *Agathobacter rectalis*, a beneficial gut commensal of the *Clostridia* class, representing a rare cross-taxon prophage-host scenario.^27^ ^,^ ^28^ Patient-3 exhibited the least donor-induced change in phage composition. Nevertheless, the selective increase in *Clostridioides* phage phiCD27 (vOTU_152), and repression of *Lacticaseibacillus*-associated prophages (vOTUs 349, 103, and 230) suggests that FFT may influence prophage dynamics, clinically relevant to CDI.

In sum, *ex vivo* phageome analyses revealed heterogeneous dynamics within the viral fraction of the recipients' microbiomes following FFT. In Patient-1 and Patient-3, donor filtrate exposure was associated with enrichment of a complete *C. difficile* prophage, raising the possibility that phage activity contributed to microbiome dynamics in these individuals. In contrast, no comparable observation was made in Patient-2, highlighting substantial inter-individual variability in phage responses to FFT. While our data do not allow us to determine the extent to which prophage induction or donor-derived phage amplification contributed to the clinical response, these effects should be interpreted alongside non-viral components of the filtrate. These may include microbiota-derived metabolites such as bile acid derivatives, short-chain fatty acids, fermentation intermediates including succinate and lactate, amino-acid-derived metabolites, and tryptophan-/indole-related molecules, all of which have been implicated in *C. difficile* growth, survival, colonization resistance and microbiome recovery.^29-33^ At the same time, the absence of consistent phage-related signals across the cohort argues against a general role for donor-derived phages as the primary driver of clinical improvement following FFT.

### Preliminary data from the literature

Based on our findings, we hypothesize that the fecal filtrates may provide therapeutic benefit in fulminant CDI, while possibly lowering the risk of rare but severe FMT-related infections. These observations align with theories proposing that a healthy stool metabolome and potentially its virome play a critical role in fecal-based treatment efficacy.^26^ ^,^ ^34^ Nonetheless, literature evidence for microbe-reduced/microbe-free preparations remains limited.

Preclinical large-animal studies suggest that similar stool preparations can confer benefits. In broiler chickens, both FMT and fecal virome transplantation (FVT) ameliorated lipopolysaccharide-induced intestinal mucosal and microbiome injury, with more favorable immune gene expression after virome transfer.^35^ Similarly, administering early postnatal bacteria-free fecal filtrate to neonatal piglets reduced the incidence of post-weaning diarrhea, suggestive for a measure of prophylactic to reduce morbidity in swine.^36^ Furthermore, studies utilizing preterm piglet models of human necrotizing enterocolitis (NEC) found that bacteria-free fecal filtrate outperformed FMT in terms of NEC prevention and safety, although the authors recommended additional evaluations before human application.^37^ ^,^ ^38^ Murine studies further support a therapeutic potential of the fecal filtrate. In antibiotic-perturbed mice, transplantation of autochthonous FVT restored the gut microbiome toward its pre-perturbation state.^39^ FVT given from lean-phenotype to obese-phenotype mice led to improved body weight and glycemic control, by changes in the gut microbiome and expression of genes associated with obesity and type-2 diabetes.^40^ ^,^ ^41^ Moreover, FVT has been linked to increased abundance of beneficial bacteria like *Akkermansia muciniphila*, as well as improvements in fertility rates.^26^ In radiation-induced intestinal injury, bacteria-free fecal filtrate transplantation promoted recovery in mice, coinciding with increased *Lachnospiraceae* family abundance and fecal short-chain fatty acid levels.^42^ However, evidence from inflammatory gut diseases is mixed. In a murine colitis model, bacteriophages derived from patients with ulcerative colitis aggravated disease severity.^43^ Complementing this, studies using *in vitro* human colon models have shown that introducing allochthonous viromes into predefined bacterial communities induced diversity shifts across multiple phyla, including *Bacteroidetes*, *Firmicutes*, and *Actinobacteria.* ^44^ Ecological changes in bacterial/viral compositions upon introducing a high-titer single-phage preparation into *in vitro* fecal fermentation systems was also described earlier.^45^

Clinical evidence remains limited but suggests a role for metabolome- and virome-mediated effects in human fecal-based therapies. In recurrent CDI, enrichment of donor-derived *Caudovirales* in recipients has been associated with successful post-FMT response.^46^ Moreover, lyophilized capsules derived from fecal supernatant with reduced bacterial content were successfully employed in recurrent CDI.^47^ In contrast, sterile fecal filtrate transplantation did not yield clinical benefit in metabolic syndrome or hepatic encephalopathy, despite measurable microbiome effects.^48^ ^,^ ^49^ To date, only two additional studies have evaluated bacteria-free fecal filtrate preparations for CDI, but in populations with substantially less severe immunosuppression than in our cohort.^26^ ^,^ ^50^ Notably, the recent multicentre, randomized, double-blinded non-inferiority trial by Kao et al. found that lyophilised sterile fecal filtrate did not establish non-inferiority compared with lyophilized donor stool for preventing recurrent *C. difficile* infection at 8 weeks, supporting the potential importance of live microorganisms in conventional FMT efficacy. However, this trial differed substantially from our study, as it evaluated lyophilized capsule-based therapy for recurrent CDI prevention.^50^ In addition, it may be hypothesized that restoration of secondary bile acid metabolism serves as a metabolomic mediator of clinical remission following FFT. In the healthy colon, primary bile acids undergo deconjugation by bacterial bile salt hydrolases (BSHs), enzymes widely distributed among major gut phyla including *Bacteroidetes*, *Firmicutes*, and *Actinobacteria.* ^51^ ^,^ ^52^ It has been documented that primary bile salts, especially taurocholate, act as potent germinants for *C. difficile* spores, whereas secondary bile salts such as deoxycholic acid and lithocholic acid inhibit spore germination and vegetative growth.^53^ ^,^ ^54^ In patients with recurrent *C. difficile* infection (rCDI), the gut microbiota is severely depleted, resulting in a collapse of secondary bile acid production, and a concomitant accumulation of primary bile acids, favoring *C. difficile* germination. Weingarden et al. demonstrated that patients with rCDI have a profoundly altered fecal bile acid profile dominated by primary bile acids, and that successful FMT results in a rapid shift toward a donor-like pattern rich of secondary bile acids. Similarly, Seekatz et al. reported that restoration of bile acid metabolism after FMT parallels the recovery of bacterial taxa involved in bile acid transformation, including members of the families *Lachnospiraceae* and *Ruminococcaceae* which harbor 7α-dehydroxylating capacity.^29^ ^,^ ^30^ ^,^ ^55^ Importantly, the regeneration of secondary bile acid pools after FMT is dependent on the re-establishment of specific functional guilds within the gut microbiome. For example, Mullish et al. showed that the recovery of BSH enzymatic activity in post-FMT stool samples was a strong predictor of clinical cure in rCDI patients, and the restoration of BSH-producing taxa such as *Bacteroides*, *Collinsella* and *Blautia* was accompanied by a reduction in taurocholic acid levels, and a reciprocal increase in secondary bile acids.^56^ Furthermore, beyond direct antimicrobial effects, secondary bile acids signal through host receptors, thereby influencing host metabolism, immune function and gut barrier integrity.^33^ ^,^ ^57^ Thus, restoration of secondary bile acid profiles may also represent an important functional readout of microbiome-associated metabolic recovery following FFT administration.

### Limitations

As there is no established gold-standard therapy for fulminant CDI in profoundly immunocompromised patients, our pilot study lacked a matched-control group. The small sample size and the use of a single donor further limits generalizability, and the establishment of definitive conclusions regarding the safety and efficacy of FFT. An additional limitation is that combination anti-CDI therapy was discontinued on the day of first FFT administration in all patients, and therefore, the relative contribution of antibiotic withdrawal and FFT to subsequent microbiome changes cannot be disentangled. Discontinuation of broad-spectrum and anti-anaerobic antimicrobial pressure may itself permit partial recovery of anaerobic taxa and broader microbiome restructuring. Accordingly, the observed post-FFT compositional changes should not be interpreted as direct evidence of a specific FFT-mediated effect. However, all patients had shown ongoing clinical deterioration despite several days of combination anti-CDI therapy before FFT, and clinical improvement occurred only after FFT administration and treatment discontinuation. Moreover, microbiome responses were heterogeneous rather than uniformly restorative, suggesting that antibiotic withdrawal alone is unlikely to fully explain the observed clinical and ecological trajectories. In addition, culture-based screening has inherent limits of detection and may not fully capture low-abundance organisms or indirect ecological effects after FFT. Nevertheless, we believe that our pilot results are hypothesis-generating and, if confirmed in larger controlled studies with dense longitudinal microbiome and metabolomic sampling, may help establish FFT as a therapeutic alternative for a vulnerable patient population with limited treatment options in the future.

## Conclusion

Fecal filtrate transplantation appears to be a feasible therapeutic option for fulminant CDI in adult patients with chemotherapy-induced aplasia.

## Supplementary Material

Supplementary material FMT_SUPPLEMENTARY_TABLE_S7.pdf

Supplementary material FMT_SUPPLEMENTARY_FILE_2.pdf

Supplementary material FMT_SUPPLEMENTARY_FILE_4.pdf

Supplementary material FMT_SUPPLEMENTARY_FILE_1.pdf

Supplementary material FMT_SUPPLEMENTARY_TABLE_S1.pdf

Supplementary material FMT_SUPPLEMENTARY_FILE_3.pdf

Supplementary material FMT_SUPPLEMENTARY_FIGURE_1.png

Supplementary material FMT_SUPPLEMENTARY_FIGURE_4.png

Supplementary material FMT_SUPPLEMENTARY_LIST_R2.docx

Supplementary material FMT_SUPPLEMENTARY_FIGURE_6.png

Supplementary material FMT_SUPPLEMENTARY_FIGURE_7.png

Supplementary material FMT_SUPPLEMENTARY_TABLE_S2.pdf

Supplementary material FMT_SUPPLEMENTARY_FILE_6.pdf

Supplementary material FMT_SUPPLEMENTARY_FIGURE_5.png

Supplementary material FMT_SUPPLEMENTARY_TABLE_S6.xlsx

Supplementary material FMT_SUPPLEMENTARY_FIGURE_2.png

Supplementary material FMT_SUPPLEMENTARY_FIGURE_3.png

## Data Availability

The data and materials that support the findings of this study are available from the corresponding author upon reasonable request. Restrictions apply to the availability of raw patient-level data due to ethical and privacy considerations.

## Abbreviations

ACG: American College of Gastroenterology
AML: acute myeloid leukemia
ASID: Australasian Society of Infectious Diseases
ASTCT: American Society for Transplantation and Cellular Therapy
ASV: amplicon sequence variant
BID: bis in die (twice daily)
CDI: *Clostridioides difficile* (*C. difficile*) infection
cdtA/cdtB: *C. difficile* binary toxin
cgMLST: core genome MLST
CLA-Ven: cladribine + cytosine-arabinoside + venetoclax
CMMoL: chronic myelomonocytic leukemia
CPM: counts per million
CRP: C-reactive protein
CT: computed tomography
CT: complex type
CTCAE: Common Terminology Criteria for Adverse Events
ECOG: Eastern Cooperative Oncology Group
ESCMID: European Society of Clinical Microbiology and Infectious Diseases
FDX: fidaxomicin
FLAG-M: fludarabine + cytosine arabinoside + filgrastim + mitoxantrone
FMT: fecal microbiota transplantation
FFT: fecal filtrate transplantation
FVT: fecal virome transplantation
GvHD: graft-versus-host disease
HSCT: hematopoietic stem cell transplantation
IDSA: Infectious Diseases Society of America
IV: intravenous
JCPG: Japanese Clinical Practice Guidelines
MDS: myelodysplastic syndrome
MLST: multilocus sequence typing
MTZ: metronidazole
NaCl: sodium chloride
NCCN: National Comprehensive Cancer Network
NEC: necrotizing enterocolitis
PCoA: principal coordinates analysis
PCT: procalcitonin
QID: quater in die (four times daily)
rCDI: recurrent *Clostridioides difficile* (*C. difficile*) infection
RT: ribotype
SOC: standard-of-care
ST: sequence type
tcdA: *C. difficile* toxin A
tcdB: *C. difficile* toxin B
Thio-Bu-Flu: thiotepa + busulphan + fludarabine
TID: ter in die (three times daily)
TIG: tigecycline
VAN: vancomycin
vOTU: viral operational taxonomic unit
WGS: whole genome sequencing
WSES: world society of emergency surgery
