# Supplementary material for: Fecal filtrate transplantation as salvage therapy for fulminant Clostridioides difficile infection in adult hematological patients during chemotherapy-induced aplasia: a pilot experience
Source: Gut Microbes. 2026 Aug 24;18(1):2718620. doi: 10.1080/19490976.2026.2718620 (PMC13523979; doi:10.1080/19490976.2026.2718620)
Supplement: Supplementary file 1 [file KGMI_A_2718620_SM9663.pdf]

MetaPhage report: MultiQC Report

Sample	vOTUs_number	vOTUs_tot_abundance	min_vOTU_length	avg_vOTU_length	max_vOTU_length	Violin_plots
4025_Clos_null_S7_L001	220.00	916,395.00	3,005.00	14,248.09	128,315.00	K_4193_prefilt_7_S34_L001_violin_plot.html")
4024_Clos_null_S8_L001	205.00	535,986.00	3,005.00	9,417.52	46,614.00	K_4193_prefilt_7_S34_L001_violin_plot.html")
K_4025_prefilt_S32_L001	558.00	16,538,701.00	3,001.00	14,057.44	157,505.00	K_4193_prefilt_7_S34_L001_violin_plot.html")
4024_pre_filt_S31_L001	1,005.00	7,931,913.00	3,005.00	13,865.72	157,505.00	K_4193_prefilt_7_S34_L001_violin_plot.html")
K_4193_prefilt_7_S34_L001	501.00	503,882.00	3,005.00	17,843.75	128,315.00	K_4193_prefilt_7_S34_L001_violin_plot.html")
K_4193_prefilt_0_S3_L001	1,294.00	2,926,012.00	3,001.00	12,779.46	157,505.00	K_4193_prefilt_7_S34_L001_violin_plot.html")
D4025_Clos_S4_L001	1,162.00	23,503,006.00	3,005.00	13,061.52	157,505.00	K_4193_prefilt_7_S34_L001_violin_plot.html")
D_P1_S10_L001	1,143.00	13,471,525.00	3,005.00	13,223.24	157,505.00	K_4193_prefilt_7_S34_L001_violin_plot.html")
D4024_Clos_S5_L001	1,083.00	28,180,897.00	3,005.00	13,588.07	157,505.00	K_4193_prefilt_7_S34_L001_violin_plot.html")
D4193_Clos_S6_L001	1,193.00	30,261,754.00	3,005.00	13,400.96	157,505.00	K_4193_prefilt_7_S34_L001_violin_plot.html")
D_P2_S11_L001	1,040.00	16,883,407.00	3,005.00	13,377.31	157,505.00	K_4193_prefilt_7_S34_L001_violin_plot.html")
D_P3_S12_L001	1,122.00	18,019,576.00	3,005.00	13,069.53	157,505.00	K_4193_prefilt_7_S34_L001_violin_plot.html")
4193_pre_7_filt_S2_L001	1,251.00	24,305,007.00	3,005.00	13,067.10	157,505.00	K_4193_prefilt_7_S34_L001_violin_plot.html")
4193_Clos_null_S9_L001	256.00	26,798,811.00	3,005.00	10,081.03	74,479.00	K_4193_prefilt_7_S34_L001_violin_plot.html")
4025_pre_filt_S30_L001	1,186.00	17,630,100.00	3,001.00	14,209.91	157,505.00	K_4193_prefilt_7_S34_L001_violin_plot.html")
K_4024_prefilt_S33_L001	539.00	25,241,436.00	3,001.00	14,920.22	157,505.00	K_4193_prefilt_7_S34_L001_violin_plot.html")
4193_pre_0_filt_S1_L001	1,376.00	11,914,779.00	3,001.00	12,557.13	157,505.00	K_4193_prefilt_7_S34_L001_violin_plot.html")
