# Supplementary material for: Fecal filtrate transplantation as salvage therapy for fulminant Clostridioides difficile infection in adult hematological patients during chemotherapy-induced aplasia: a pilot experience
Source: Gut Microbes. 2026 Aug 24;18(1):2718620. doi: 10.1080/19490976.2026.2718620 (PMC13523979; doi:10.1080/19490976.2026.2718620)
Supplement: Supplementary file 2 [file KGMI_A_2718620_SM9665.pdf]

	Target	Clinical specimen	Type of test	Test name	Product code	Manufacturer
Virology	Rotavirus / Adenovirus	Stool	Antigen test	Combi K-Set (Rota+Adeno) with collection set	K-1504/TB	Corisbio
	Calicivirus	Stool	Antigen test	Rida Quick Norovirus	N1402	r-biopharm
	Enteroviruses	Stool	PCR	in-house method	n.r.	n.r.
	HAV	Stool	PCR	AltoStar® HAV RT-PCR Kit 1.5	AS0241543	Altona
	HEV	Stool	PCR	HEV ELITe MGB Kit, HEV ELITe Standard, HEV – ELITe Positive Control	RTS130ING, STD130ING, CTR130ING	EliTechGroup
	SARS-CoV-2	Stool	PCR (not validated for stool)	Allplex™ SARS-CoV-2/FluA/FluB/RSV Assay	RV10349Z	Seegene Inc.
Bacteriology	*Shigella sp., Salmonella sp.*	Stool	Conventional culturing	XLD agar	43563	bioMérieux SA
	Campylobacter sp.	Stool		Campylobacter blood-free selective agar CCDA	M1064	Clinichem kft.
	Yersinia sp.	Stool		Chromagar Y. enterocolitica	201484	Mast diagnostica
	Screening for MDR bacterial carriage (MRSA, VRE, ESBL, MPAE, MACI, CPE)	Stool	Conventional culturing	Eosin Methylene Blue EMB Agar	43081	bioMérieux SA
				Columbia agar 5% with sheep blood	43049	bioMérieux SA
				Chromid VRE	43004	bioMérieux SA
				Meat extract broth	mex40010	Biolab
				Selenite-f broth	42099	bioMérieux SA
				Mast ESBL set(D52c)	171055	Mast diagnostica
				Imipenem IPM 10ug	CT0455B	Oxoid
				Mueller hinton E 90 mm	413824	bioMérieux SA
	Helicobacter pylori	Stool	Antigen test	CerTest H. pylori Card	P820001V	Certest
	Clostridioides difficile	Stool	Antigen test / toxin test	C. Diff. Check Complete	T30550C	Techlab
Parastiology	Giardia sp. / Cryptosporidium sp.	Stool	Antigen test	GIARDIA/CRYPTOSPORIDIUM Quick Check	T30407	Techlab
	Entamoeba sp.	Stool	Antigen test	Rida Quick Entamoeba	N1703	r-biopharm
	Ova and enteral parasites/protozoons (most notably for Giardia, Cryptosporidium, Isospora, Cyclospora, Microsporidia, Entamoeba sp.)	Stool	Direct and iodine-stained wet mount + acid-fast bacilli stain	Conventional techniques	n.r.	n.r.
			Flotation, sedimentation, cyst enrichment, larval translocation methods	Conventional techniques	n.r.	n.r.
Mycology	Candida sp.	Stool	Conventional culturing	Sabouraud Gent. Chloramp. Agar	43651	bioMérieux SA
Complex	Biofire GI panel (Adenovirus F40/41, Astrovirus, Norovirus GI/GII, Rotavirus A, Sapovirus I, II, IV, and V, Campylobacter (C. jejuni, C. coli, C. upsaliensis), Clostridioides (Clostridium) difficile (toxin A/B), Plesiomonas shigelloides, Salmonella, Yersinia enterocolitica, Vibrio (V. parahaemolyticus, V. vulnificus, V. cholerae), Vibrio cholerae, Enteroaggregative E. coli (EAEC), Enteropathogenic E. coli (EPEC), Enterotoxigenic E. coli (ETEC lt/st), Shiga-like toxin-producing E. coli (STEC stx1/stx2), E. coli O157, Shigella / Enteroinvasive E. coli (EIEC), Cryptosporidium, Cyclospora cayetanensis, Entamoeba histolytica, Giardia lamblia)	Stool	Multiplex PCR	BIOFIRE® FILMARRAY® Gastrointestinal (GI) Panel	RFIT-ASY-0116, RFIT-ASY-0104	bioMérieux SA
Blood serology	anti-HAV IgM	Blood serum	CLIA	HAV IgM	310180	DiaSorin/Liaison
	anti-HAV total	Blood serum	CLIA	anti-HAV	310170	DiaSorin/Liaison
	anti-HEV IgM + IgG	Blood serum	ELISA	recomWell HEV test	MG-5005, MG-5004	Mikrogen
	HBsAg	Blood serum	CLIA	HBsAg Quant	310250	DiaSorin/Liaison XL Murex
	anti-HBc IgM + IgG	Blood serum	CLIA	anti-HBc	310130	DiaSorin/Liaison
	anti-HBs IgG	Blood serum	CLIA	anti-HBc	311220	DiaSorin/Liaison XL Murex
	anti-HCV IgG	Blood serum	CLIA	HCV Ab	310240	DiaSorin/Liaison XL Murex
	HIV1/2 Ab+Ag	Blood serum	CLIA	HIV Ab/Ag	310260	DiaSorin/Liaison XL Murex
	Treponema pallidum Ab	Blood serum	CLIA	Treponema Screen	310840	DiaSorin/Liaison
	Entamoeba histolytica IgG	Blood serum	ELISA	NovaLisa® Entamoeba histolytica IgG	ENTG0140	Novatec Immundiagnostica
	Strongyloides stercoralis IgG	Blood serum	ELISA	NovaLisa® Strongyloides	STRO0690	Novatec Immundiagnostica
