# Supplementary material for: Fecal filtrate transplantation as salvage therapy for fulminant Clostridioides difficile infection in adult hematological patients during chemotherapy-induced aplasia: a pilot experience
Source: Gut Microbes. 2026 Aug 24;18(1):2718620. doi: 10.1080/19490976.2026.2718620 (PMC13523979; doi:10.1080/19490976.2026.2718620)
Supplement: Supplementary file 4 [file KGMI_A_2718620_SM9667.pdf]

SUPPLEMENTARY FILE 1. The stool donor evaluation process used at our center

A) Donor eligibility during the pre-screening (questionnaire + clinical assessment):

1. **A person <u>can be considered</u> a donor if:**

   a) They are an adult between 18–45 years of age (preferably as young as possible)

   b) They have no known documented disease or health impairment

   c) They have a normal BMI (18–25 kg/m^2)

   d) They follow a normal mixed diet

   e) They do not regularly take medications, dietary supplements or herbal products

   f) They do not live in the same household as the recipient

   g) They are willing to comply with the donation protocol and to donate *pro bono*

2. **<u>Absolute contraindications</u> to stool donation at the pre-screening phase:**

   a) Known chronic infection or ongoing acute infection at the time of examination

   b) Known gastrointestinal or neuro-psychiatric disease

   c) Diarrhea, melena, or hematochezia within the past 6 months

   d) Any history of illicit drug use

   e) Any history of risky sexual contact (sexual contact with an unknown person, a sex worker, a drug addict or with a person suffering from a known chronic infection or other sexually transmitted disease)

   f) Any history of solid organ or hematopoietic stem cell transplantation

   g) Any history of or active onco-hematological malignancy

   h) Needle-stick injury, piercing, tattoo, or ear piercing within the past 12 months

   i) Transfusion of any blood product within the past 12 months

   j) Travel to a tropical or subtropical country within the past 12 months

   k) Immunomodulatory, immunosuppressive, chemotherapy, or systemic antimicrobial therapy within the past 12 months or ongoing

   l) Employment in healthcare or animal husbandry

   m) Any abnormality detected during a face-to-face evaluation and physical examination with a physician that raises the possibility of a serious ongoing disease or one that currently precludes donation

Donor safety: If during pre-screening, none of the absolute contraindications to stool donation are present in the applicant, the evaluation (*screening*) of ideal candidates may be initiated. The evaluation of multiple potential donors can be started simultaneously.

B) Blood and stool screening of eligible potential donors after pre-screening:

1. The microbiological examinations, the range of pathogens to be tested, and the methods are listed in **Supplementary file 2.**
2. **Timing of stool sampling:**

   a) Stool parasite/protozoa examinations must be performed from stool collected on 3 consecutive days, with at least 24 hours between collections, using 3 separate fresh (within 2 hours), warm samples (cooling or freezing is prohibited).

   b) Stool bacteriological, virological, and mycological examinations are carried out from the last collected stool sample.

   c) Blood serological tests are performed together with routine laboratory tests on the last day.
3. **Routine laboratory tests, in addition to microbiological examinations:**

   a) Complete blood count + reticulocyte count, fragmentocyte count, immature platelet fraction

   b) Serum Na, K, Ca (total and ionized), Mg, Pi, Cl, HCO_3, Osmolality

   c) Serum creatinine, urea, eGFR (CKD-EPI)

   d) Serum GOT, GPT, GGT, ALP, LDH, CK

   e) Blood glucose, serum bilirubin, serum uric acid, total protein, albumin, globulin, protein electrophoresis + immunofixation, serum immunoglobulins, serum CRP, TSH

   f) INR, aPTT, TT, fibrinogen
4. **Absolute contraindications to stool donation at the screening phase:**

   a) An infection is detected during microbiological testing that makes stool donation impossible.

   b) A laboratory finding emerges that indicates a previously unknown/undiagnosed disease.

Donor safety: For the donor’s safety, any infection or other health abnormality identified during screening will, of course, be disclosed to the donor, and they will be referred to the appropriate healthcare provider.

C) Donation procedure:

1. The entire stool passed by the selected donor on the same day is collected under contamination-free conditions.
2. From the collected donation, the microbiological examinations detailed above are repeated; until then, the donation is stored frozen at –80 °C (–112 °F).
3. Donated stool samples are quarantined until all test results are confirmed negative.

Donor safety: Candidates who successfully complete all stages of pre-screening and screening are accepted as donors. A donor may not donate stool if a relevant change in health status has occurred since the pre-screening and screening (e.g., onset of fever). Donors are required to undergo repeated blood and stool screenings, typically every 8-12 weeks, to ensure continued safety.

REFERENCE WORK FOR THE DONOREVALUATION PROCESS

Aira A et al. Recommendations for stool donor selection for fecal microbiota transplant. Consensus document endorsed by the Catalan Society of Digestology, Catalan Society of Infectious diseases and Clinical Microbiology and the GEMBIOTA group from Spanish Society of Infectious Diseases and Clinical Microbiology. Enfermedades infecciosas y microbiologia clinica (English ed). 2022; 40: 142-146. https://doi.org/10.1016/j.eimce.2021.12.001

Battipaglia et al. Fecal microbiota transplantation before or after allogeneic hematopoietic transplantation in patients with hematologic malignancies carrying multidrug-resistance bacteria. *Haematologica*. 2019; 104 (8): 1682-1688. https://doi.org/10.3324/haematol.2018.198549

Bibbo et al. Fecal Microbiota Transplantation: Screening and Selection to Choose the Optimal Donor. *J Clin Med*. 2020; 9 (1757): 1-14. https://doi.org/10.3390/jcm9061757

Cammarota G et al. European consensus conference on faecal microbiota transplantation in clinical practice. Gut. 2017; 66: 569-580. https://doi.org/10.1136/gutjnl-2016-313017

Haifer C et al. Australian consensus statements for the regulation, production and use of faecal microbiota transplantation in clinical practice. Gut. 2020; 69: 801-810. https://doi.org/10.1136/gutjnl-2019-320260

Ianiro G et al. Reorganisation of faecal microbiota transplant services during the COVID-19 pandemic. Gut. 2020; 69: 1555-1563. https://doi.org/10.1136/gutjnl-2020-321829

European Committee on Organ Transplantation. Guide to the quality and safety of tissues and cells for human application, 5th edition. EDQM. 2022.
