# Supplementary material for: Fecal filtrate transplantation as salvage therapy for fulminant Clostridioides difficile infection in adult hematological patients during chemotherapy-induced aplasia: a pilot experience
Source: Gut Microbes. 2026 Aug 24;18(1):2718620. doi: 10.1080/19490976.2026.2718620 (PMC13523979; doi:10.1080/19490976.2026.2718620)
Supplement: Supplementary file 5 [file KGMI_A_2718620_SM9668.pdf]

GUIDELINE	DOI	PHARMACOLOGICAL TREATMENT			NON-PHARMACOLOGICAL TREATMENT	
		Oral	Rectal enema	Intravenous	Surgical	FMT
**ACG (2021)**	10.14309/ajg.0000000000001278	VAN 500 mg QID (first 48–72 hours) then VAN 125 mg QID	+VAN 500 mg QID (in ileus)	Combination with MTZ 500 mg TID (can be considered)	Total colectomy with end ileostomy or diverting loop ileostomy with colonic lavage and intraluminal VAN	Consider FMT, when refractory or patient is deemed apoor surgical candidate
**ASID (2016)**	10.1111/imj.13027	1st line: VAN 125 mg QID 3rd line: FDX 200 mg BID	2nd line: ±VAN 500 mg QID	2nd line combination: MTZ 500 mg TID 3rd line: TIG 50 mg BID (100 mg loading dose)	Indications for surgery: 1. Toxic megacolon or bowel perforation 2. Deterioration despite 1st and 2nd line medical therapy Total colectomy or diverting loop ileostomy with intraluminal VAN	Recommended only after SOC failure
**ASTCT (2022)**	10.1016/j.eclinm.2025.103371	VAN 500 mg QID	±VAN 500 mg QID (in ileus)	Combination with MTZ 500 mg TID (can be considered)	No details	No details
**BSG/HIS (2024)**	10.1016/j.jhin.2024.03.001	No details	No details	No details	No details	Consider FMT earlier than after second CDI recurrence for patients with severe, fulminant or complicated CDI who are not responding to antibiotic therapy
**ESCMID (2021)**	10.1016/j.cmi.2021.09.038	VAN 125 mg QID or FDX 200 mg BID	When oral administration not possible	MTZ: routine additionnot recommended TIG: may be considered (50 mg BID, 100 mg loading dose)	Partial/total colectomy or loop ileostomy	Consider FMT, when refractory
**JCPG (2022)**	j.jiac.2021.12.011	1st line: VAN 125mg QID 2nd line: FDX 200 mg BID or VAN 500 mg QID	VAN 500 mg QID	2nd line combination: MTZ 500 mg TID ± bezlotoxumab	No details	No details
**SHEA/IDSA (2021)**	10.1093/cid/ciab549	VAN 500 mg QID	±VAN 500 mg QID (in ileus)	Combination with MTZ 500 mg TID	No details	No details
**WSES (2019)**	10.1186/s13017-019-0228-3	VAN 500 mg QID	±VAN 500 mg QID	Combination with MTZ 500 mg TID ± IVIG	Total colectomy or diverting loop ileostomy with colonic lavage	No details

ACG: *American College of Gastroenterology*, ASID: *Australasian Society of Infectious Diseases*, ASTCT: *American Society for Transplantation and Cellular Therapy*, BID: *bis in die* (twice daily), ESCMID: *European Society of Clinical Microbiology and Infectious Diseases*, FDX: fidaxomicin, FMT: fecal microbiota transplantation, IDSA: *Infectious Diseases Society of America*, JCPG: *Japanese Clinical Practice Guidelines*, MTZ: metronidazole, QID: *quater in die* (four times daily), SOC: standard-of-care, TID: *ter in die* (three times daily), TIG: tigecycline, VAN: vancomycin, WSES: *World Society of Emergency Surgery*

Table 1. Pharmacological and non-pharmacological treatment strategies for fulminant *C. difficile* infection, according to current international guidelines
