# Supplementary material for: Fecal filtrate transplantation as salvage therapy for fulminant Clostridioides difficile infection in adult hematological patients during chemotherapy-induced aplasia: a pilot experience
Source: Gut Microbes. 2026 Aug 24;18(1):2718620. doi: 10.1080/19490976.2026.2718620 (PMC13523979; doi:10.1080/19490976.2026.2718620)
Supplement: Supplementary file 6 [file KGMI_A_2718620_SM9669.pdf]

TIDieR

Fecal filtrate transplantation for fulminant *Clostridioides difficile* infection in adult hematological patients during chemotherapy-induced aplasia

**Why:**	Patients at the highest risk for neutropenic infections due to chemotherapy-induced aplasia include those with acute leukaemia receiving induction chemotherapy and individuals undergoing allogeneic hematopoietic stem cell transplantation (HSCT). In these cohorts, chemotherapy frequently induced severe mucositis, further increasing the likelihood of invasive infections. Moreover, *C. difficile* is the leading infectious cause of persistent diarrhea in patients undergoing treatment for active hematologic malignancies. (...) Currently, only a single international guideline focusing on hematologic patients is available for the treatment of CDI. According to this guideline, therapeutic options for fulminant CDI are limited. In general, the usage of fecal microbiota transplantation (FMT) in hematologic patients is not specifically addressed, whereas in non-hematologic patients, FMT is usually recommended when conservative therapeutic options have been exhausted or surgical intervention is not feasible.According to a recent update of the 10th *European Conference On Infections In Leukaemia* (ECIL-10), more data are required on the use of FMT in profoundly neutropenic patients, particularly in patients with fulminant CDI.Nonetheless, aversion to FMT in both hematologic and non-hematologic patients has been linked to the occurrence of rare but severe adverse reactions, such as sepsis or aspiration pneumonia.
**What (material):**	The study intervention consisted of treatment with fecal filtrate transplantation (FFT). Details on the preparation of the FFT is described in the Methods section of the manuscript, under the subheading "Fecal filtrate preparation". Details on the Donor screening and recruitment are given in Supplementary files 1 and 2. The donor evaluation was conducted in accordance with international guidelines, and included a detailed medical history assessment, as well as laboratory and microbiological examinations.
**What (procedures):**	In all patients, the prepared filtrates were administered in two separate doses, with a ~5-day interval between. For thawing, a blood warmer was used with a heating time of 1 hour (SAHARA-III/230V, Sarstedt). The supernatant was delivered via a single-lumen 14-Fr 120 cm Levin-type nasogastric tube (Changzhou Weite Medical Equipment Co.), and as premedication, 10 mg of intravenous metoclopramide was administered. The process took an average of 6 minutes, followed by flushing the tube with 30 ml of sterile 0.9% NaCl solution. At the end of the first hour of observation, the nasogastric tube was removed.
**Who provided:**	The procedure of preparing, handling and administering the FFT as an intervention was done by our study personnel, consisting of board certified hematologists and infectologists. All medical doctors involved in the study worked in full accordance with the protocol provided in the manuscript.
**How (mode of delivery; individual or group):**	The FFT was delivered as described earlier in Point 4.
**Where:**	The FFT was delivered in the patient's own hospital room. All other features are detailed in the Methods section of our manuscript, under the subheading "Method of administration".
**When and how much:**	The total volume of the administered supernatant was approximately 220 ml for Patient-1 and Patient-2, and 240 ml for Patient-3. (...) In all patients, the prepared filtrates were administered in two separate doses, with a ~5-day interval between.

Tailoring: The amount of stool used to prepare a complete supernatant graft for a recipient was determined individually, based on the recipient’s body weight. Specifically, 2 grams of stool were processed per kilogram of body weight, corresponding to 120 g of raw graft for Patient 1 and Patient 2, and 130 g for Patient 3.

Modification: There were no unforeseen modifications to the intervention during the course of the study.

How well (planned): FFT was well-tolerated by the participants, without any immediate complaints.

How well (actual): As the intervention was given directly by study personnel without the aid of the patients, no protocol deviations were documented.
