# Supplementary material for: Fecal filtrate transplantation as salvage therapy for fulminant Clostridioides difficile infection in adult hematological patients during chemotherapy-induced aplasia: a pilot experience
Source: Gut Microbes. 2026 Aug 24;18(1):2718620. doi: 10.1080/19490976.2026.2718620 (PMC13523979; doi:10.1080/19490976.2026.2718620)
Supplement: Supplementary file 7 [file KGMI_A_2718620_SM9675.pdf]

	shannon_entropy	faith_pd	pielou_evenness	observed_features
Patient_1_pre_fft	4,083306702	4,818389162	0,74793623	44
Patient_1_post_fft_d14	4,451080904	5,685842917	0,792753504	49
Patient_1_post_fft_d30	7,066945546	14,04014638	0,92987192	194
Patient_2_pre_fft	3,81884705	5,040722459	0,856352867	22
Patient_2_post_fft_d14	6,014485721	6,008114424	0,933499685	87
Patient_3_pre_fft	5,9510727	9,728350426	0,848791871	129
Patient_3_post_fft_d14	5,228705904	9,028347306	0,803452812	91
Patient_3_post_fft_d30	4,330552411	2,665720953	0,793224041	44
Donor	6,460131816	7,667067813	0,952630754	110
