# Supplementary material for: Fecal filtrate transplantation as salvage therapy for fulminant Clostridioides difficile infection in adult hematological patients during chemotherapy-induced aplasia: a pilot experience
Source: Gut Microbes. 2026 Aug 24;18(1):2718620. doi: 10.1080/19490976.2026.2718620 (PMC13523979; doi:10.1080/19490976.2026.2718620)
Supplement: Supplementary file 8 [file KGMI_A_2718620_SM9676.pdf]

Supplementary File 5. Detailed description of the next-generation sequencing

Samples were sequenced using Illumina Nextera XT (paired-end, 2×150 bp; 10M reads/sample). Raw reads were processed using Metaphage v0.3.2, a comprehensive pipeline for viral metagenome analysis that includes quality control (fastp and seqScreener) (**Supplementary Table 1.),** host/read decontamination (Kraken2 with Krona visualization) (**Supplementary Figures 1 and 2.**), *de novo* assembly (Megahit) and assembly quality assessment (QUAST) [15]. The assembled contigs were screened using four viral detection tools: VIBRANT, VirSorter, Phigaro, and VirFinder (**Supplementary Figure 3.**). Contigs with predicted phage origins were clustered into vOTUs using CD-HIT-EST at 95% nucleotide identity, with a word size of 9 and alignment coverage threshold of 0.85. Only vOTUs with predicted completeness >50% (assessed using CheckV) were retained for downstream analysis (**Supplementary Tables 2. and 3.)** [16]. Read mapping to vOTUs was performed using BowTie2, and coverage calculated with BamToCov. More than 90% of raw reads passed quality filtering across all samples. Beta-distances between samples were calculated using Bray-Curtis dissimilarity metric, visualized on PCoA analysis plot (**Supplementary Figure 4.**). Contig abundance was normalized to account for library size and contig length, resulting in Counts Per Million (CPM) values for each vOTU (**Supplementary Table 2.**). Contig abundance values, referred to as CPM throughout the manuscript, were calculated using TPM-style normalization: raw read counts were first divided by contig length in kilobases, then scaled by the total sum of these length-normalized counts to one million per sample. Criteria for enrichment classification to identify phage elements enriched in recipient samples or derived from the donor was to: 1. show presence in the donor filtrate (CPM >1); 2. be absent in control groups (CPM <0.1) and 3. have increased abundance in the experimental recipient mix (**Supplementary Tables 4. and 5.**). Recipient-derived enrichment followed the same rules, with reversed roles. This classification allows identification of both donor-contributed and recipient-propagated phage populations potentially associated with therapeutic outcomes, including indirect suppression of *C. difficile* via modulation of the host microbiota or direct lytic activity. Suppression, or induction of prophage production associated with the presence of donor filtrate was determined in consideration of fold changes due to different library sizes and calculating significant deviation from baseline CPM values (**Figure 2.**).
