# Supplementary material for: Fecal filtrate transplantation as salvage therapy for fulminant Clostridioides difficile infection in adult hematological patients during chemotherapy-induced aplasia: a pilot experience
Source: Gut Microbes. 2026 Aug 24;18(1):2718620. doi: 10.1080/19490976.2026.2718620 (PMC13523979; doi:10.1080/19490976.2026.2718620)
Supplement: Supplementary file 9 [file KGMI_A_2718620_SM9672.docx]

**All supplementary files are available for download at**: https://figshare.com/s/07c34a58fe5aeadda9a3

**SUPPLEMENTARY FILES**

**Supplementary File 1**. The stool donor evaluation process used at our center

**Supplementary File 2.** Blood and stool microbiological screening of eligible potential donors

**Supplementary File 3.** The completed TIDieR Checklist

**Supplementary File 4.** The completed CONSORT Statement

**Supplementary File 5.** Jaccard distance matrix (in .qza file)

**Supplementary File 6.** Detailed description of the next-generation sequencing

**SUPPLEMENTARY TABLES**

**Supplementary Table 1.** Pharmacological and non-pharmacological treatment strategies for fulminant *C. difficile* infection, according to current international guidelines

**Supplementary Table 2.** Alpha diversities within the patient cohort, calculated by the Shannon entropy, Faith’s Phylogenetic Diversity, Pielou's Evenness, and the number of observed features by ASV richness

**Supplementary Table 3.** Beta diversity distances within the patient cohort, calculated using the Bray-Curtis metric

**Supplementary Table 4.** Unweighted UniFrac distance matrix

**Supplementary Table 5.** Weighted UniFrac distance matrix

**Supplementary Table 6.** Relative abundances of bacterial taxa within the patient cohort

**Supplementary Table 7.** MetaPhage and MultiQC Report

**Supplementary Table 8.** vOTU Completeness Table

**Supplementary Table 9.** Taxonomy Table

**Supplementary Table 10.** RRC Table

**Supplementary Table 11.** CountCPM Table

**SUPPLEMENTARY FIGURES**

**Supplementary Figure 1.** Alpha rarefaction analysis of the 16S rRNA gene amplicon dataset. Curves are shown for observed features, representing ASV richness, and Shannon diversity, reflecting both richness and evenness. Sequencing depth ranged from 4,293 to 13,363 reads per sample. Core diversity analyses were performed at a sampling depth of 4,290 reads per sample, selected to retain all samples.

**Supplementary Figure 2.** Jaccard beta-diversity analysis of the 16S rRNA gene amplicon dataset.

**Supplementary Figure 3.** Weighted UniFrac beta-diversity analysis of the 16S rRNA gene amplicon dataset.

**Supplementary Figure 4.** Beta-diversity analysis of bacteriophage communities using Principal Coordinates Analysis (PCoA). The plot shows beta-diversity patterns of viral communities across patient samples, donor filtrates, and their mixtures. Distances were calculated using the Bray-Curtis dissimilarity metric

**Supplementary Figure 5.** Quality control report

**Supplementary Figure 6.** Ratio of reads of taxa per sample as predicted by Kraken2

**Supplementary Figure 7.** Number of vOTUs predicted per phage contig miner tools
